# Integrated genomic analysis defines molecular subgroups in dilated cardiomyopathy and identifies novel biomarkers based on machine learning methods

**DOI:** 10.3389/fgene.2023.1050696

**Published:** 2023-02-07

**Authors:** Ling-Fang Ye, Jia-Yi Weng, Li-Da Wu

**Affiliations:** ^1^ Changzhi People’s Hospital, Changzhi, Shanxi, China; ^2^ Department of Cardiology, The Affiliated Suzhou Hospital of Nanjing Medical University, Suzhou Municipal Hospital, Gusu School, Nanjing Medical University, Suzhou, China; ^3^ Nanjing Medical University, Nanjing, China

**Keywords:** dilated cardiomyopathy, WGCNA, molecular subgroups, lasso algorithm, SVM-RFE algorithm

## Abstract

**Aim:** As the most common cardiomyopathy, dilated cardiomyopathy (DCM) often leads to progressive heart failure and sudden cardiac death. This study was designed to investigate the molecular subgroups of DCM.

**Methods:** Three datasets of DCM were downloaded from GEO database (GSE17800, GSE79962 and GSE3585). After log2-transformation and background correction with “*limma*” package in R software, the three datasets were merged into a metadata cohort. The consensus clustering was conducted by the “*Consensus Cluster Plus*” package to uncover the molecular subgroups of DCM. Moreover, clinical characteristics of different molecular subgroups were compared in detail. We also adopted Weighted gene co-expression network analysis (WGCNA) analysis based on subgroup‐specific signatures of gene expression profiles to further explore the specific gene modules of each molecular subgroup and its biological function. Two machine learning methods of LASSO regression algorithm and SVM-RFE algorithm was used to screen out the genetic biomarkers, of which the discriminative ability of molecular subgroups was evaluated by receiver operating characteristic (ROC) curve.

**Results:** Based on the gene expression profiles, heart tissue samples from patients with DCM were clustered into three molecular subgroups. No statistical difference was found in age, body mass index (BMI) and left ventricular internal diameter at end-diastole (LVIDD) among three molecular subgroups. However, the results of left ventricular ejection fraction (LVEF) statistics showed that patients from subgroup 2 had a worse condition than the other group. We found that some of the gene modules (pink, black and grey) in WGCNA analysis were significantly related to cardiac function, and each molecular subgroup had its specific gene modules functions in modulating occurrence and progression of DCM. LASSO regression algorithm and SVM-RFE algorithm was used to further screen out genetic biomarkers of molecular subgroup 2, including *TCEAL4*, *ISG15*, *RWDD1*, *ALG5*, *MRPL20*, *JTB* and *LITAF*. The results of ROC curves showed that all of the genetic biomarkers had favorable discriminative effectiveness.

**Conclusion:** Patients from different molecular subgroups have their unique gene expression patterns and different clinical characteristics. More personalized treatment under the guidance of gene expression patterns should be realized.

## Introduction

Dilated cardiomyopathy (DCM) is the most common type of cardiomyopathy and a leading cause of death in the cardiovascular field, which is characterized by enlargement of the ventricle and reduced cardiac function (Fatkin et al., 2019). DCM can develop into severe congestive heart failure progressively and threaten the survival of patients. Although tremendous progress has been made in the treatment field of DCM in the past decades, the morbidity and mortality of DCM still remain high (Jefferies and Towbin, 2010). At present, the etiology and pathogenesis of DCM are still unclear. Most of DCM cases were thought to be sporadic, but at least 40%–60% of DCM cases are now found to be familiar. Pedigree analysis showed that most of families with DCM had autosomal dominant inheritance, while a few had autosomal recessive inheritance, mitochondrial inheritance and X-linked inheritance. It is of clinical significance to identify the underlying the genetic mechanisms of DCM, which will improve the prognosis of patients with DCM.

With the development of gene sequencing technologies, the public gene expression profile databases, such as TCGA database and GEO database, provide us an opportunity to better understand the underlying genetic mechanisms of DCM. Bioinformatics analysis can identify the differentially expressed genes (DEGs) of DCM and uncover the specific biological functions of DEGs, which plays a crucial role in developing clinical therapeutic measures and new drugs (Cordero et al., 2008). Xiao et al. used dataset of DCM (GSE3585) downloaded from GEO database to screen out the DEGs of DCM patients compared with control group and identified the hub genes (*CTGF*, *IGFBP3*, *SMAD7*, *INSR*, *CTGF*, *IGFBP3*) significantly related to DCM by establishing protein-protein interaction (PPI) network (Zhang et al., 2017). In addition, Huang *et al.* also analyzed the DCM heart tissue samples from the GEO database (GSE79962) using weighted gene co-expression network analysis (WGCNA) method, and identified gene modules that are related to the progression of DCM (Kang et al., 2020).

Molecular classification was first proposed in various cancer researches to reveal the heterogeneity between patients with the same tumor, shifting tumor classification from traditional morphology to molecular features-based molecular typing. Considering patients in different molecular subgroups often have different clinical manifestations and prognosis, molecular classification is helpful in judging prognosis and guiding treatment of diseases (Travaglino et al., 2020a; Travaglino et al., 2020b; Naso et al., 2021). In recent years, more and more researchers have focused on the molecular classification among chronic diseases rather than tumors, such as idiopathic pulmonary fibrosis (IPF), coronary artery disease (CAD) and hepatitis B virus (HBV) infection (Ainali et al., 2012; Zhang et al., 2021a; Zhang et al., 2021b). CAD is a leading cause of death in cardiovascular field. To investigate the molecular features of patients with CAD in different molecular subgroups, Peng et al. also performed molecular subgroups analysis and classified 352 patients with CAD into three molecular subgroups based on datasets downloaded from GEO database. They found that patients in different molecular subgroups of CAD not only showed different gene expression patterns, but also different clinical characteristics (Ainali et al., 2012). As a complex inherited disease similar to cancer, DCM also exhibited clinical heterogeneity. Nevertheless, the molecular subgroups of DCM have not been reported. Therefore, we carried out this work to conduct molecular classification of patients with DCM, looking for specific gene modules in each molecular subgroup and exploring the relationship between each molecular subgroup and clinical features. Many studies have analyzed the gene expression profiles related to DCM. However, most of the previous studies screened out differentially expressed genes (DEGs) between DCM patients and control individuals, but ignored the existed differences in gene expression profiles among DCM patients. In the present study, we further classified DCM patients into molecular subgroups based the gene expression patterns, and revealed that patients from different subgroups exhibited different clinical characteristics. Artificial intelligence (AI) is a new technical science that researches and develops theories, methods, technologies and application systems for simulating, extending and expanding human intelligence (Ghazal et al., 2022). Medicine is one of the earliest applications of AI, including disease diagnosis and the selection of the best surgical procedures (Goyal et al., 2022). Machine learning is an important branch of artificial intelligence and has been widely used in screening characteristic genes and risk factors of diseases (Dai et al., 2022; Liu et al., 2022; Wu et al., 2022). We also used machine learning methods to screen characteristic genes in subgroups in an attempt to correlate gene expression profiles with clinical features in patients with DCM.

## Methods

### Data collection

Three gene expression datasets of DCM were downloaded from GEO database (http://www.ncbi.nlm.nih.gov/geo/) (Barrett et al., 2013) *via* the “*GEO query*” package in R software (version 4.1.1, http://r-project.org/) (Davis and Meltzer, 2007), including GSE17800 (Liu et al., 2022), GSE79962 (Dai et al., 2022), and GSE3585 (Barrett et al., 2013). GSE17800 was performed on the GPL570 platform and included heart tissue samples from 40 DCM patients and eight control individuals (Ameling et al., 2013). GSE79962 was performed by GPL6244 platform and included nine DCM samples and 11 control samples (Matkovich et al., 2017). GSE3585 was based on the platform GPL96, which includes heart tissue samples from seven DCM patients and five control individuals (Barth et al., 2006). The detailed characteristics of datasets was shown in Table 1.

**TABLE 1:** Characteristics of the datasets included in the analysis.

GEO ID	Platform	Citation	Region	Control	DCM
GSE41177	GPL570; Affymetrix Human Genome U133 Plus 2.0 Array	Ameling et al. (2013)	Greifswald, Germany	8	40
GSE79962	GPL6244; Affymetrix Human Gene 1.0 ST Array	Matkovich et al. (2017)	St. Louis, USA	11	9
GSE3585	GPL96; Affymetrix Human Genome U133A Array	Barth et al. (2006)	Heidelberg, Germany	5	7

GEO: Gene Expression Omnibus; DCM: dilated cardiomyopathy.

### Data processing

Gene expression matrices of GSE17800, GSE79962, and GSE3585 were established by R software. Then, we employed the “*limma*” package to conduct log2-transformation and background correction, and merged three datasets into a metadata cohort for further analysis (Davis and Meltzer, 2007). Considering the integrated datasets were based on different platforms and different experiment conditions, it is of significance to remove the batch effect. The “*SVA*” package was adopted for removing batch effects (Yeh et al., 2013). Moreover, each gene expression value from different batches were adjusted by the normalization procedure of “central standardization,” also known as “mean centering” using “*Combat*” package. Finally, the “*ggplot2*” package was adopted to conduct principal component analysis (PCA) and draw PCA-plot based on the top two principal components in PCA (Ito and Murphy, 2013).

### Consensus clustering

The consensus clustering of DCM samples from GSE17800, GSE79962, and GSE3585 was conducted by the “*Consensus Cluster Plus*” package (Wilkerson and Hayes, 2010). We set 10 as the maximum value of cluster groups. The consistency score (greater than 0.7 in all clusters) and cumulative distribution function (CDF) was used to determine the number of cluster groups.

### Comparing the clinical features among molecular groups

Clinical characteristics were also obtained by “*GEO query*” package (Subramanian et al., 2007; Nidheesh et al., 2017). To obtain the difference of clinical features among different molecular subgroups, the clinical characteristics of the three subgroups were compared in detail. We adopted the Pairwise Wilcoxonʼs rank-sum test to investigate whether there were differences in age, BMI, LVEF and LVIDD among three subgroups. The analysis of variance for age, molecular subgroup and their interaction was also conducted to validate whether the factor of molecular subgroup classification is an independent indicator that can predict severity of DCM.

### WGCNA analysis

WGCNA method is an effective tool to identify co-expression modules related to specific biological function (Langfelder and Horvath, 2008). We adopted WGCNA according to the subgroup‐specific signatures to determine potential gene modules that can represent the functions of each molecular subgroup of DCM. In the scale-free network, the best soft-threshold power was determined by maximal *R* ^2^. Moreover, we used the average method and the dynamic method to conduct hierarchical clustering analysis. After merging of similar modules, the module classification of genes were ultimately established. Correlation analysis between WGCNA modules and clinical characteristics was also performed using Spearmanʼs method.

### Enrichment analysis

The “*clusterProfler*” package (Wu et al., 2021) was used to perform GO and KEGG pathway enrichment analysis among different modules to further investigate the biological meaning of different modules and its roles in occurrence and progression of DCM. We downloaded the gene group reference of KEGG pathway from MSigDB database (Kanehisa and Goto, 2000; Kanehisa et al., 2019). The filter was set as *p*-value < 0.05 in KEGG analysis.

### Identification of biomarkers based on machine learning methods

We adopted two machine learning methods of LASSO regression algorithm and SVM-RFE algorithm to screen out biomarkers of molecular subgroup of DCM. “*glmnet*” package was employed to conduct LASSO regression algorithm, which is a linear regression model and widely used to screen characteristic genes or elements most closely related to disease occurrence (Zhang et al., 2014). SVM-RFE is another machine learning algorithm, which has also been widely used for classification and regression analysis. We used SVM-RFE algorithm based on “*e107*” package to identify genes with high discriminative power (Leavey et al., 2018). Genes identified by both algorithms were eventually selected as biomarkers.

### Evaluation of discriminative power of the biomarkers

We created receiver operating characteristic (ROC) curve by the “*pROC*” package, and area under curve (AUC) value was adopted to determine the discriminative power.

## Results

### Removal of batch effect

**FIGURE 1:**
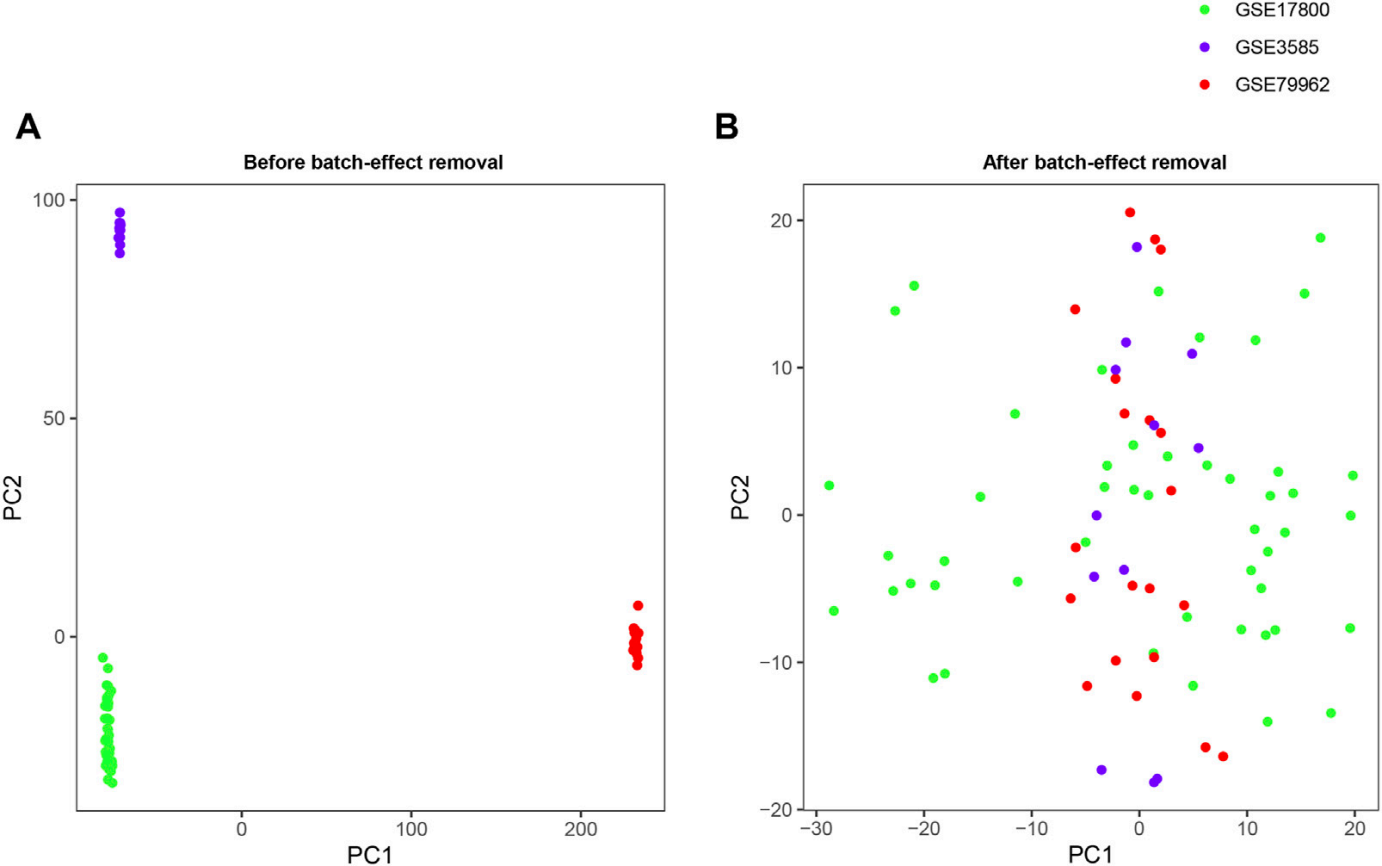
PCA plots of the gene expression datasets. The points of the PCA plots visualize the samples based on the top two PC (PC1 and PC2) without **(A)** and with **(B)** the removal of batch effect between GSE17800, GSE79962 and GSE3585. PCA, Principal component analysis; PC, principal components.

The detailed characteristics of the datasets included in the analysis, including GSE17800, GSE79962 and GSE3585, was shown in Table 1. A total of 11,779 genes were jointly detected by both microarray platforms of the dataset. Principal component analysis (PCA) was performed to validate whether the batch effect among the datasets included in this study was successfully removed. PCA‐plot was drawn based on the top two principal components (PCs) in PCA. Before the process of batch effect removing, heart samples from patients with DCM were clustered by batches, indicating that there was significant batch effect caused by different platforms and different experiment conditions among the datasets (Figure 1A). In addition, the distribution range of specimens on the horizontal (PC1) and vertical (PC2) axes is 100 and 200, respectively, with a large variation rate. After removing of batch effect between GSE17800, GSE79962 and GSE3585, including samples of controls and patients with DCM, the PCA‐plot based on PCA of the normalized meta-cohort data revealed that the batch effect between GSE17800, GSE79962, and GSE3585 was clearly removed. Of note, the batch effect between samples of controls and patients with DCM was also removed (Figure 1B).

### Consensus clustering of DCM cases

**FIGURE 2:**
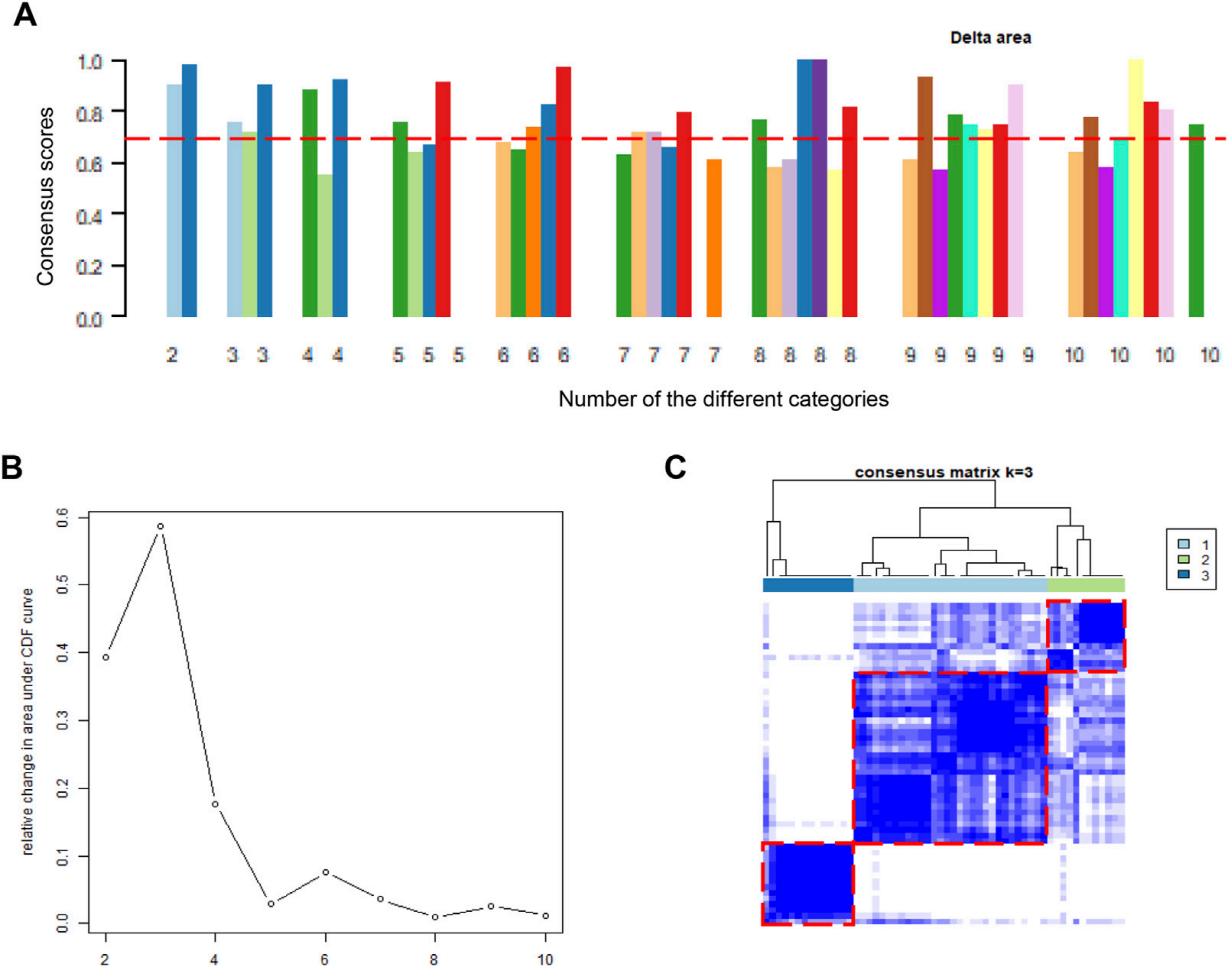
Consensus clustering analysis based on gene expression profiles of DCM patients. **(A)**. The barplots of consistency scores of each cluster; **(B)**. The CDF scores of the different categories; **(C)**. The heatmap represents the consensus matrix with cluster count of 3, which was determined by the CDF scores and consensus scores of subgroups. DCM, dilated cardiomyopathy; CDF, cumulative distribution function.

After the batch effect was successfully removed, the merged dataset was employed to conduct molecular subgroup analysis by consensus clustering. The cluster consensus score of each subgroup was higher than 0.7 only in the three categories (Figure 2A). In addition, CDF curve showed that the CDF score was the largest in the three categories (Figure 2B). Both evidences suggested that three molecular subgroups were more robust than others in DCM patients. Therefore, heart tissue samples would be clustered into three molecular subgroups according to the consistency score and the CDF curve. In the consensus matrix, we observed that there is a high similarity of gene expression patterns within each molecular subgroup (Figure 2C). Ultimately, we adopted consensus clustering algorithm to divide 56 heart tissue samples from patients with into three molecular subgroups based on the gene expression patterns.

### The differences of clinical characteristics in the three molecular subgroups

**FIGURE 3:**
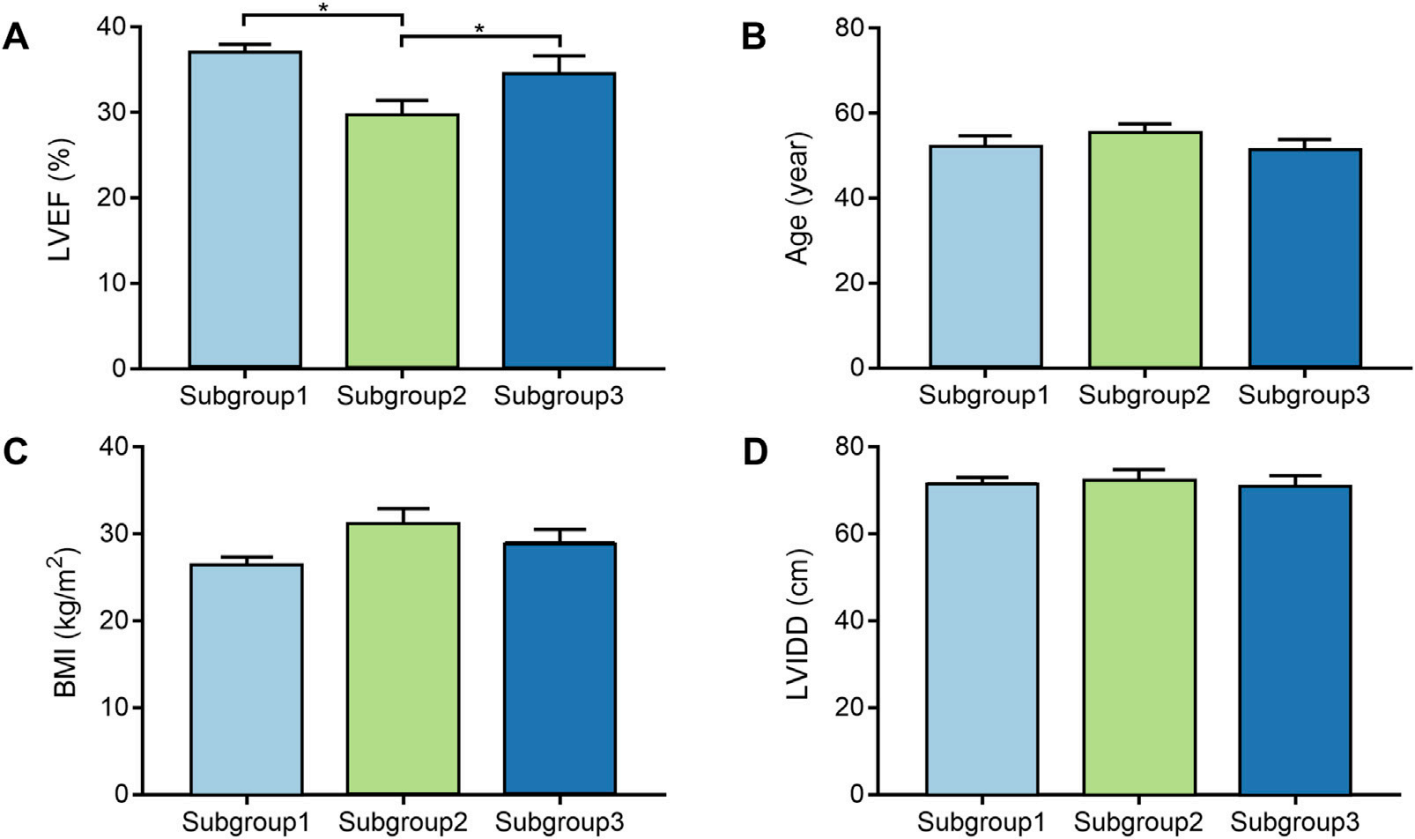
The comparison of clinical characteristics among the different molecular subgroups. **(A)**. Box plot displays LVEF of each subgroup; **(B)**. Box plot displays age of each subgroup; **(C)**. Box plot displays BMI of each subgroup; **(D)**. Box plot displays LVIDD of each subgroup. BMI, body mass index; LVEF, left ventricular ejection fraction; LVIDD, left ventricular internal diameter at end-diastole.

DCM cases in subgroup 1, subgroup 2, and subgroup 3 had different gene expression patterns. To further investigate the clinical characteristics of three groups, the age, BMI, LVEF, and LVIDD were analyzed in detail in DCM cases from GSE17800 dataset. We found that patients in subgroup 2 had lower LVEF than patients in subgroup 1 and subgroup 3 with statistical difference (Figure 3A). However, the results of age, BMI, and LVIDD statistics showed that there was no significant difference among three groups (Figures 3B–D). As a result, not only did gene expression differs, but the severity of the disease also varied among three subgroups of DCM cases. As shown in Table 2, the analysis of variance (ANOVA) on age and our molecular classification was performed, indicating that the molecular classification in the present study was an age-independent indicator for the severity of DCM.

**TABLE 2:** Analysis of variance for classification of subgroups, age, and their interactions.

	Df	Sum square	Mean square	F value	Pr (>F)
Subgroup	2	411.4	205.6	6.03	0.006^∗∗^
Age	1	0.5	0.53	0.016	0.902
Subgroup and age interaction	2	32.3	16.13	0.473	0.63
Residuals	34	1159.8	34.11		

Df: degree of freedom. Significant codes: “∗∗∗” 0.001, “∗∗” 0.01, “∗” 0.05.

### WGCNA analysis

**FIGURE 4:**
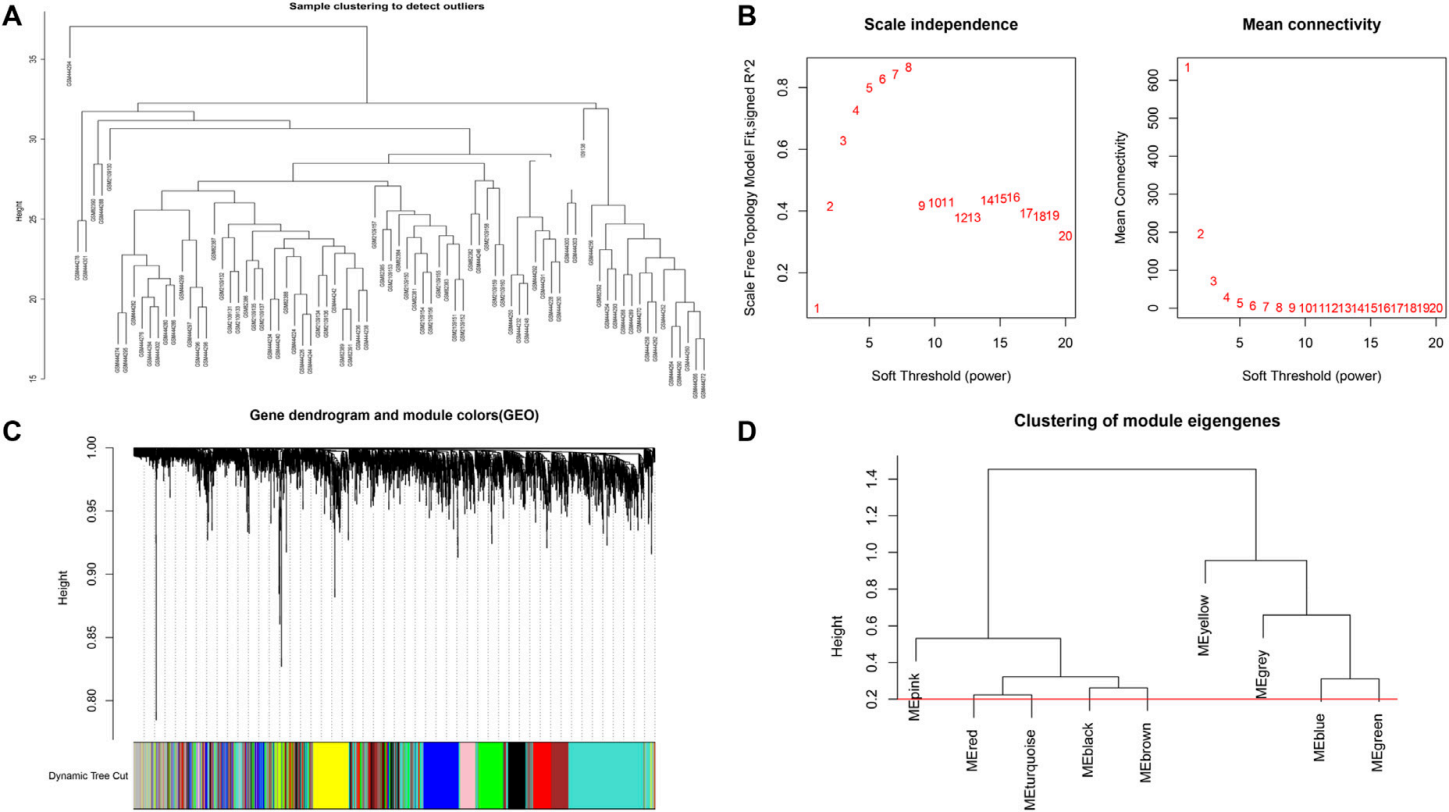
Sample clustering and network construction of the weighted co-expressed genes. **(A)** Clustering dendrogram heart tissue samples from patients with DCM and control individuals. **(B)** the scale-free index and the mean connectivity for various soft-thresholding powers. **(C)** Dendrogram clustered based on a dissimilarity measure. Gene expression similarity is assessed by a pair-wise weighted correlation metric and clustered based on a topological overlap metric into modules. Each color below represents one co-expression module, and every branch stands for one gene. **(D)** Cluster dendrogram of modules.

**FIGURE 5:**
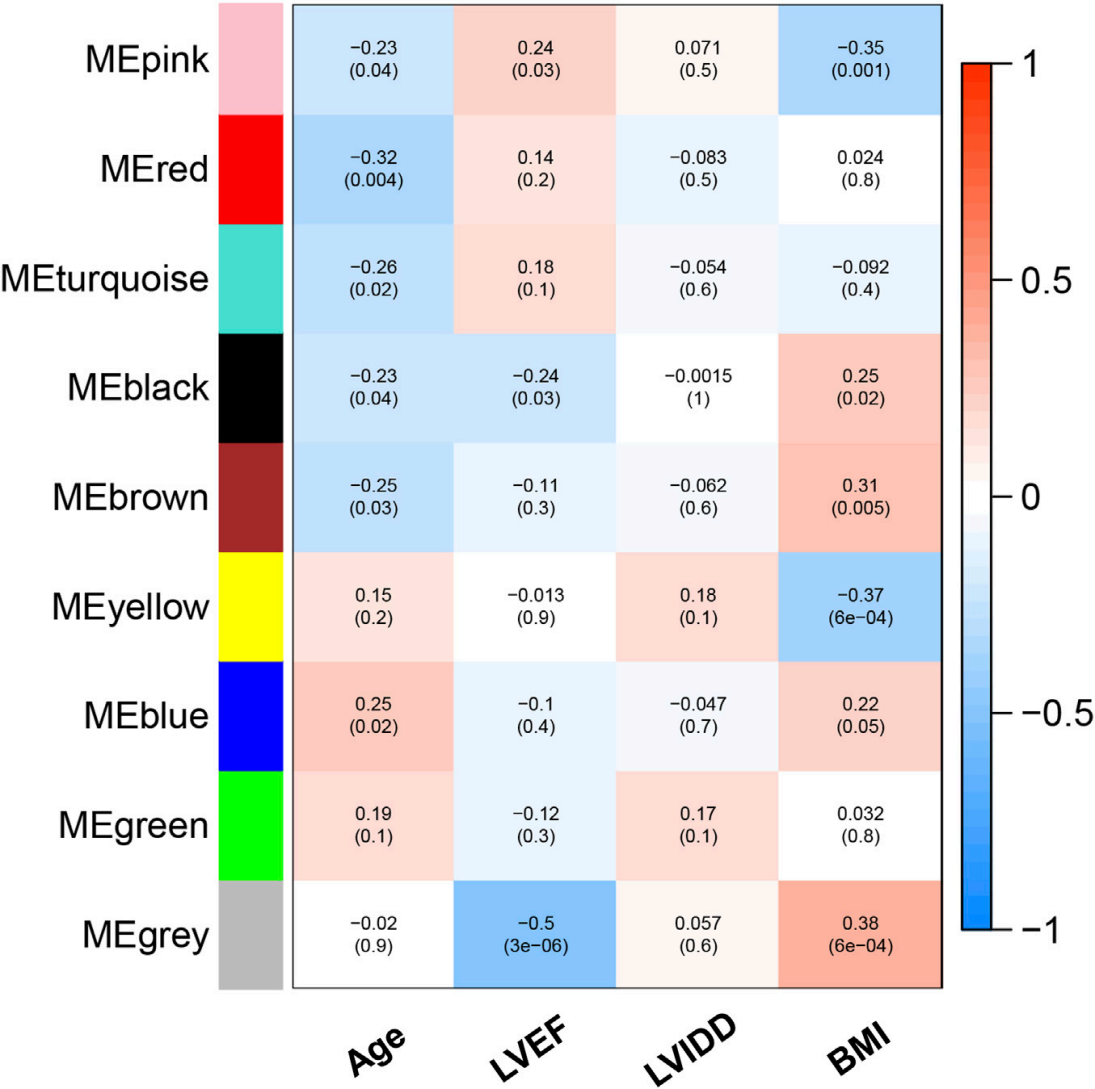
Heatmap of the correlation between modules and clinical features of patients with DCM.

**FIGURE 6:**
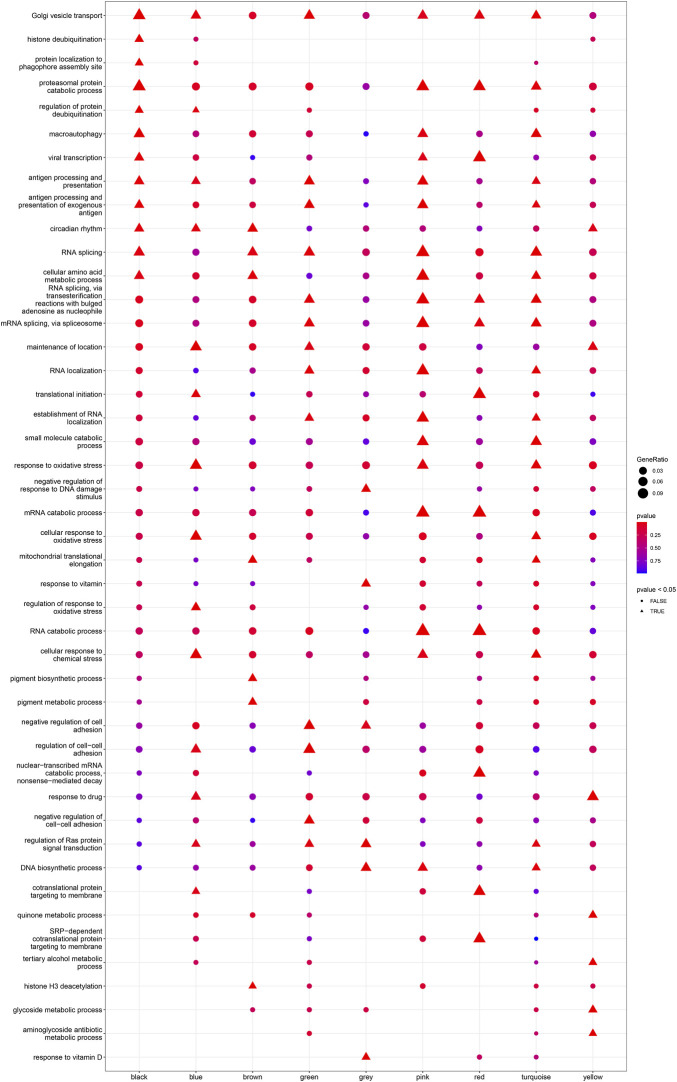
Heatmap of the enriched biological processes in GO analysis for each WGCNA module.

**FIGURE 7:**
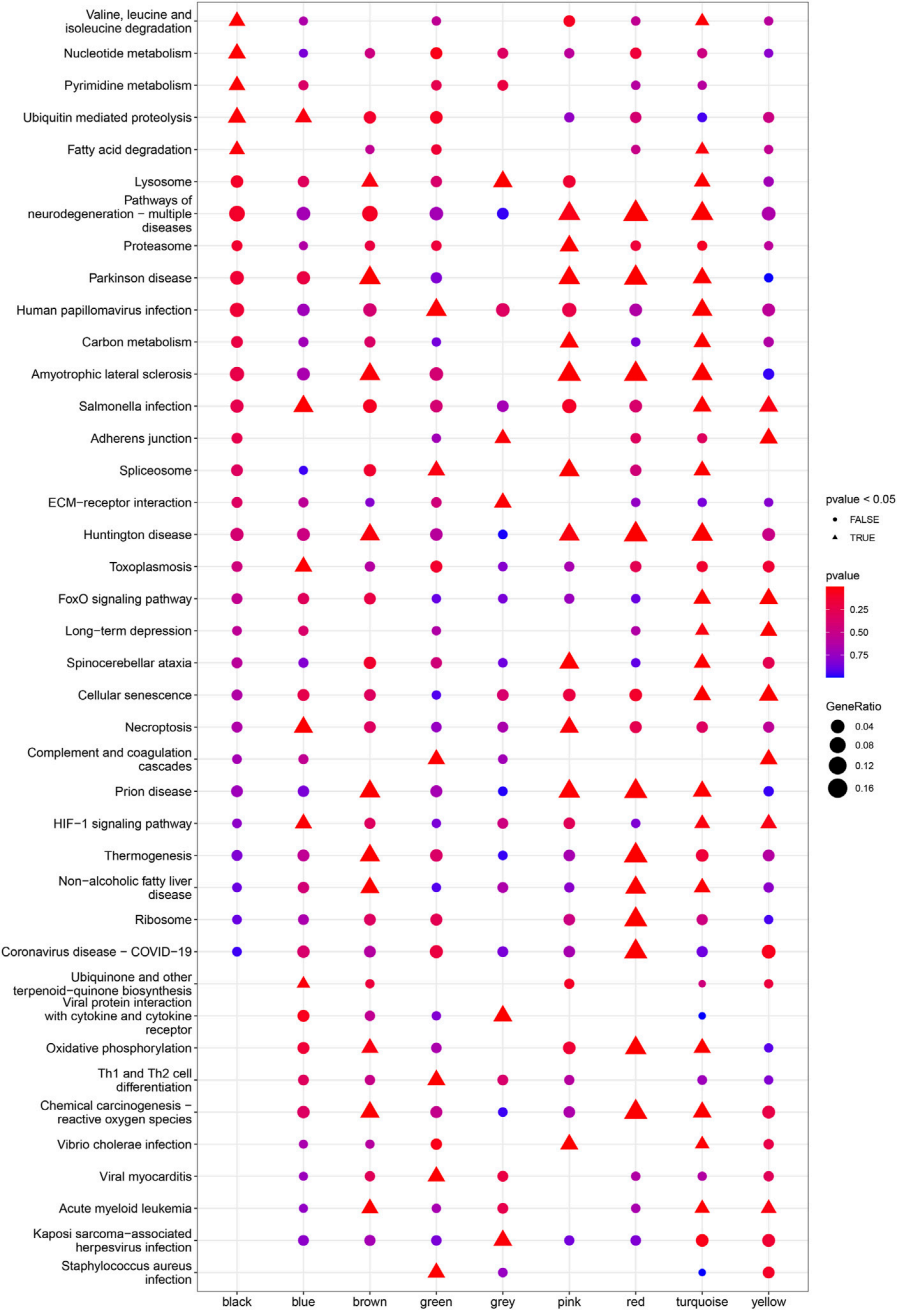
Heatmap of the enriched pathways in KEGG analysis for each WGCNA module.

Based on Pairwise differential expression analysis, we identified 605, 697, and 1,557 specific differentially expressed genes in subgroups 1, subgroups 2, and subgroups 3 compared with other subgroup (Benjamin-Hochberg adjusted *p* < 0.05, absolute difference of mean > 0.2) (Table 3). We also compared the gene expression profile of each molecular subgroup with that of control individuals. There was 1,236, 1,388, and 2,617 differentially expressed genes in subgroups 1, subgroups 2, and subgroups 3 compared with the control individuals (Table 3). To further reveal the differences in gene expression patterns and the resulting functional differences among molecular subgroups of DCM, WGCNA was performed based on the specific differentially expressed genes in each group. We carried out WGCNA analysis based on topological overlaps and scale-free network and created a hierarchical clustering tree based on the dynamic-hybrid cut (Figure 4A). According to the results of scale-free topology criterion, we selected 8 as the soft-thresholding power (*R* ^2^ = 0.89; Figure 4B). Ultimately, a total of nine co-expressed modules were identified for further research. Figure 4C shows the cluster dendrogram of the modules and the clustering of module eigengenes was shown in Figure 4D. Figure 5 shows the identified nine WGCNA modules, of which the corresponding subgroups are shown in Table 3. To further study the relationship between WGCNA modules and clinical features of patients with DCM, the correlation coefficients between WGCNA models and clinical features were calculated. As shown in Figure 5, age was correlated positively with module blue, and negatively correlated with module brown, module black, module turquoise, module red and module pink. LVEF was positively correlated with module pink, and negatively corelated with module black and module grey. BMI was positively corelated with module grey, module blue, module brown, and module black, and negatively corelated with module pink and module yellow. These results show that the WGCNA modules was associated with clinical features of patients with DCM. Moreover, we performed GO functional enrichment analysis based on the genes in different WGCNA modules. Figure 6 shows the biological process terms enriched in different modules. The abscissa represents the elder brother module, and the ordinate represents the item of functional enrichment analysis. A triangle means statistically significant. The enriched terms in cellular component and molecular function are shown in Supplementary Figures S1, S2. Detailed results of GO enrichment analysis were shown in Supplementary Tables S1–S3. We also conducted KEGG pathway analysis and identified pathways enriched in different WGCNA modules (Figure 7). Detailed results of KEGG enrichment analysis were shown in Supplementary Table S4. Above all, these results of enrichment analysis demonstrated each molecular subgroup had its specific functional gene modules that could function in modulating DCM onset or progression.

**TABLE 3:** The number of differentially expressed genes by case-control and case-case comparisons and weighted gene co-expression analysis modules in each subgroup.

Subgroups	The specific genes were compared with the normal group	The specific genes were compared with each subgroup	Modular
Subgroup 1	1236	605	Red and yellow
Subgroup 2	1388	697	Black, blue, green and grey
Subgroup 3	2617	1557	Pink, turquoise and brown

### Identification of biomarkers based on machine learning algorithms

**FIGURE 8:**
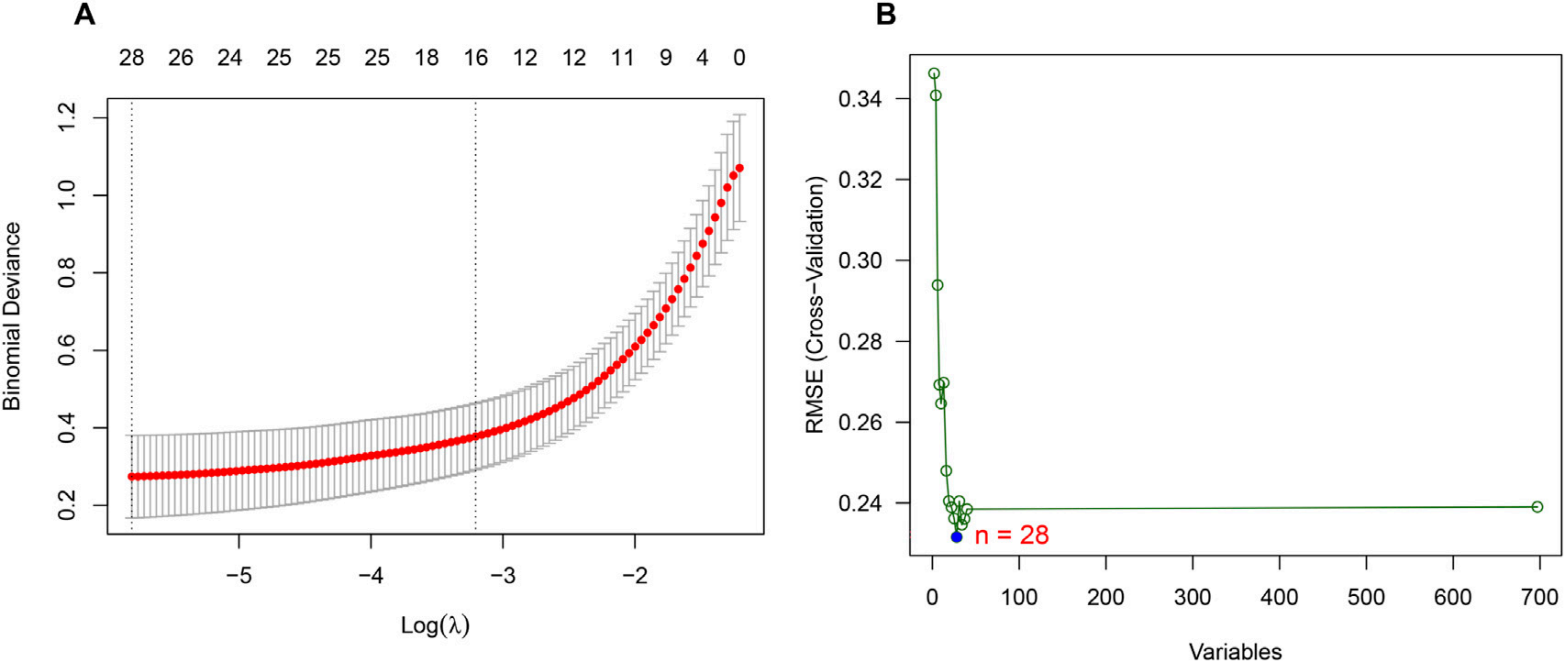
Identification of biomarkers of molecular subgroup 2 using machine learning algorithms. **(A)** Identification of biomarkers of molecular subgroup 2 *via* LASSO algorithm; **(B)** Identification of biomarkers of molecular subgroup 2 *via* SVM-RFE algorithm.

**FIGURE 9:**
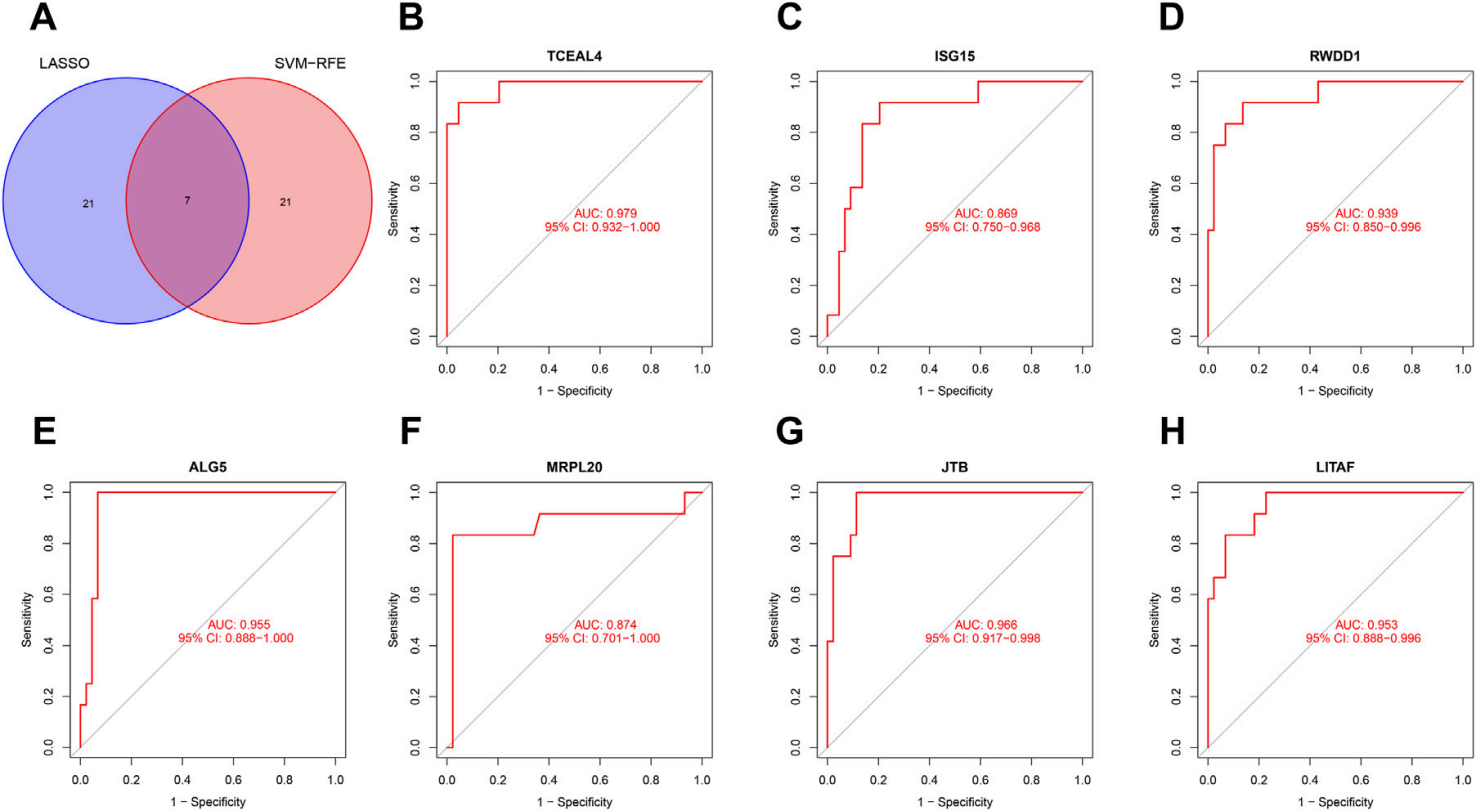
Evaluation of the effectiveness of the biomarkers. **(A)** Venn plot of the overlapping genes identified by the LASSO algorithm and SVM-RFE algorithm. **(B–H)** ROC curves of *TCEAL4*, *ISG15*, *RWDD1*, *ALG5*, *MRPL20, JTB,* and *LITAF*. ROC, receiver operating characteristic.

Considering the patients in subgroup 2 had more severe condition, two machine learning algorithms of LASSO regression and SVM-RFE algorithm were adopted to screen out biomarkers. According to the specific differentially expressed genes in subgroup 2, we screened out 28 key gene significantly related to molecular classification using LASSO algorithm (Figure 8A). In addition, 28 genes were identified as biomarkers based on the SVM-RFE algorithm (Figure 8B). The seven overlapping genes, including *TCEAL4*, *ISG16, RWDD1, ALG5, MRPL20, JTB* and *LITAF*, were finally selected as biomarkers (Figure 9A). All of the DEGs of subgroup 2 with detailed *p*-value and adjust *p*-value was shown in Supplementary Table S5.

### Diagnostic effectiveness of biomarkers

**FIGURE 10:**
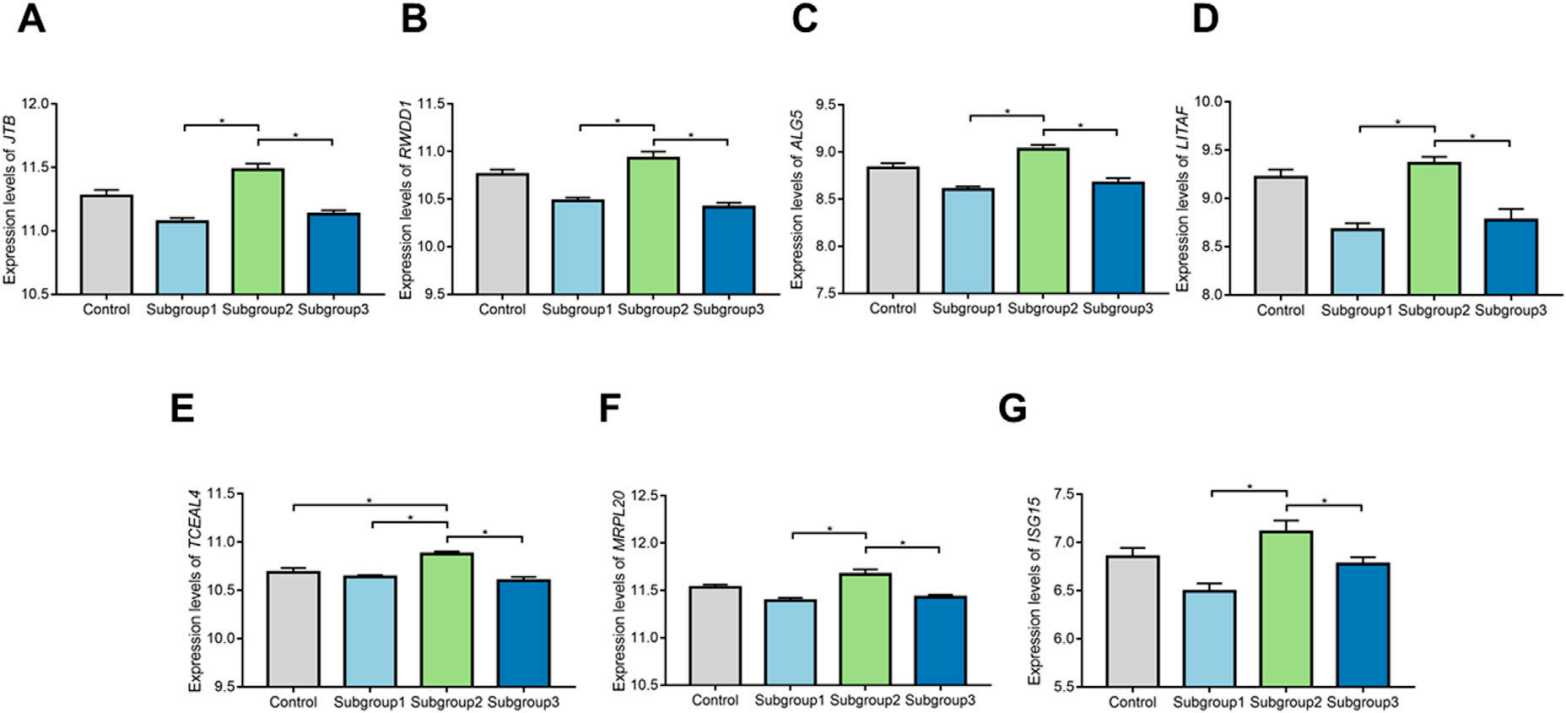
The comparison of expression levels of the biomarkers among control group and different molecular subgroups. **(A–G)** Expression levels of TCEAL4, ISG15, RWDD1, ALG5,MRPL20, JTB, and LITAF among control group and different molecular subgroups.*p < 0.05.

ROC curve was adopted to evaluate the diagnostic effectiveness of biomarkers of subgroup 2. The results of ROC curve indicated that all of the biomarkers have a favorable diagnostic effectiveness in discriminating DCM cases in subgroup 2, with an AUC of 0.979 (95% CI 0.932–1.000) in *TCEAL4*, AUC of 0.869 (95% CI 0.750–0.968) in *ISG15*, and AUC of 0.939 (95% CI 0.850–0.996) in *RWDD1,* AUC of 0.955 (95% CI 0.888–1.000) in *ALG5,* AUC of 0.874 (95% CI 0.701–1.000) in *MRPL20*, AUC of 0.966 (95% CI 0.917–0.998) in *JTB* and AUC of 0.953 (95% CI 0.888–0.996) in *LITAF* (Figures 9B–H). The expression levels of the biomarkers among different molecular subgroups were shown in Figures 10A–G.

## Discussion

In this study, three gene expression profiles of heart tissue samples from patients with DCM and control individuals from GEO database were analyzed in detail. For the first time, we merge the three datasets as a metadata cohort and successfully clustered the DCM cases into three molecular subgroups according to the gene expression profile of DCM. The consensus clustering process based on CDF score and cluster consensus score guaranteed that our molecular subgroup classification was robust. Furthermore, significant correlation between clinical conditions and molecular subgroups was observed. Patients in subgroup 2 had lower LVEF comparing with the other two subgroups. In addition, molecular subgroups-specific functional modules and pathways were also analyzed through WGCNA method. These results taken together showed that the molecular classification of DCM was associated with clinical features of patients with DCM and patients in different molecular subgroups should receive personalized treatment.

Molecular subgroup classification based on gene expression patterns has provided great help for clinical diagnosis and treatment, especially in the field of cancer research. Zhang et al. (2014) reported that the stem‐like signatures were significantly activated in patients with colon cancer from molecular subtype C. In recent years, more and more researchers have focused on the molecular classification among chronic diseases rather than tumors. For example, IPF is one of the idiopathic interstitial pneumonias with high mortality and morbidity. Zhang et al. (2021a) conducted a molecular subgroups analysis for patients with IPF according to gene expression profiles, and revealed the potential molecular features of different types of IPF. CAD is a leading cause of death in cardiovascular field. To investigate the molecular features of patients with CAD in different molecular subgroups, Peng et al. also performed molecular subgroups analysis and classified 352 patients with CAD into three molecular subgroups based on datasets downloaded from GEO database. They found that patients in different molecular subgroups of CAD not only showed different gene expression patterns, but also different clinical characteristics (Ainali et al., 2012). At present, the hepatitis B virus (HBV) infection is a public health threat worldwide. Patients infected with HBV in different molecular subgroups showed significantly differences in clinical features, such as degree of liver fibrosis and liver index. Of note, the immune cells infiltration in liver tissue samples from patients with HBV of different are also different (Zhang et al., 2021b). Understanding the gene expression patterns of diseases, especially inherited diseases and studying the clinical characteristics of different molecular subtypes are very important for the precise treatment of each patient. Moreover, psoriasis, pre‐eclampsia, Alzheimerʼs disease and myelodysplastic syndrome were also found association between the clinical variables and transcriptional differences or subtypes (Aibar et al., 2016; Leavey et al., 2018). These studies provide a deeper understanding of diseases and indicate the significance of precise medicine. In the present study, we collected gene expression datasets of DCM from GEO database and conducted an integrated bioinformatics analysis, aiming to uncover the molecular subgroups according to genes expression patterns.

In particular, patients in subgroup 2 tended to have a more serious condition than patients from subgroup 1 and subgroup 3. The results of age, BMI, and LVIDd statistics showed that there was no significant difference among three groups. Therefore, DCM patients should be distinguished by the molecular classification and receive more personalized treatment.

Compared to previous studies, the functional modules and pathways identified by WGCNA method were also connected with specific molecular subgroup of DCM (Zhou et al., 2020; Huang et al., 2021; Li et al., 2021). We found that the specific differential expression genes in subgroup 2 were mostly in the black, blue, green and grey WGCNA module. Considering the black module had a significant negative correlation with LVEF, the enrichment analysis of black module demonstrated that valine, leucine and isoleucine degradation signaling pathway, nucleotide metabolism signaling pathway and ubiquitin mediated proteolysis signaling pathway may contribute to the negative correlation with cardiac function. The change of metabolism is an important feature of DCM. Optimizing myocardial energy metabolism is one of the important means to treat DCM (Mak et al., 2021). Of note, branched chain amino acids (BCAAs) are collectively referred to as leucine, valine and isoleucine. BCAAs can be regarded as one of the most important nutritional supplements and are the most characteristic energy source for the oxidation and utilization of myocardial amino acids. Although BCAAs accounts for only 2% of myocardial ATP production, it plays an important role in regulating insulin pathway and mammalian rapamycin like target protein (mTOR) signaling pathway (Jo et al., 2022). In addition, BCAAs can continuously activate mTOR signal and damage insulin signal transduction through insulin receptor substrate, and abnormal BCAAs metabolism can cause the accumulation of BCAAs metabolites and eventually lead to insulin resistance (Cuomo et al., 2022). Studies have shown that eating a mixture rich in BCAAs can prolong the average life span of mice and increase mitochondrial biogenesis in mouse myocardium and skeletal muscle (Valerio et al., 2011). However, the increase of plasma BCAAs level in patients is considered to be an early predictor of the development of DCM. The accumulated BCAAs can activate mTOR signal and accelerate the occurrence and development of myocardial hypertrophy (Caragnano et al., 2019). Protein phosphatase PPC2m and branched-chain alpha-ketoacid dehydrogenase (BCBDK) are important targets to improve BCAA metabolism, which is crucial for BCAA oxidation and promote BCAAs oxidation. The risk of heart failure in PPC2m knockout mice was significantly increased. Enhancing BCAAs oxidation and or reducing the level of BCAA in blood have cardioprotective effects in heart failure. In addition, BCBDK inhibitor BT2 can improve the oxidation capacity of BCAA in heart failure, reduce the accumulation of BCAA, and reduce the infarct area of cardiac ischemia reperfusion injury (Li et al., 2017). Nucleotide is the basic structural unit of genetic material nucleic acid and has a variety of biological functions. In addition to being the raw material for nucleic acid synthesis, it also constitutes energy substances, such as ATP, GTP, CTP, etc., (Barvík et al., 2017). Nucleotide is also involved in metabolism and physiological regulation, for example, cAMP is an important second messenger substance in the body and participates in signal transduction (Mani, 2022). In view of the important physiological significance of nucleotide, its abnormal situation in the process of metabolism often causes serious consequences. In recent years, a series of genetic diseases, including DCM, caused by abnormal nucleotide metabolism have been found (Pant et al., 2018). Ubiquitination refers to the process in which ubiquitins (a class of low molecular weight proteins) classify proteins in cells under the action of a series of special enzymes, select target proteins from them, and modify the target proteins specifically (Kolla et al., 2022). DCM are associated with cardiac remodeling, where the ubiquitin-proteasome system (UPS) holds a central role. Different levels of UPS components, E3 ligases, and UPS activation markers were observed in myocardial tissue from control individuals and patients affected by DCM, suggesting differential involvement of the UPS in the underlying pathologies (Shukla and Rafiq, 2019). Therefore, Attention to the role of metabolic abnormalities in dilated cardiomyopathy is important to identify therapeutic targets for patients with different molecular pressure groups. We also screened out biomarkers of molecular subgroup 2, including *TCEAL4*, *ISG15*, *RWDD1*, *ALG5*, *MRPL20*, *JTB*, and *LITAF*, based on two machine learning methods of LASSO regression and SVM-RFE algorithm. However, the accuracy of its predictions requires further validation in a larger population and roles of the biomarkers in DCM still need to further investigate. A limitation of this study should be noted. The development of DCM is a complex process, although a total of 56 participants were included, the input data might still be insufficient to identify and validate biomarkers. In addition, the 56 participants included in the study came from various regions with different genetic variation, diet, physical activity and so on. Therefore, the conclusions in the present study still need more external validations.

## Conclusion

In conclusion, our results showed that, through molecular classification, more detailed disease characteristics and its relationship with clinical features of patients with DCM should be noticed. In addition, patients in different molecular subgroups should receive a more personalized treatment. Similar to molecular classification in cancer, more populations are needed to conduct further validation, moreover, future research in DCM should also introduce multi-omics data to reveal more precise molecular subgroups of DCM.

## Data Availability

The datasets presented in this study can be found in online repositories. The names of the repository/repositories and accession number(s) can be found in the article/Supplementary Material.
