# Supplementary material for: Integrated genomic analysis defines molecular subgroups in dilated cardiomyopathy and identifies novel biomarkers based on machine learning methods
Source: Front Genet. 2023 Feb 7;14:1050696. doi: 10.3389/fgene.2023.1050696 (PMC9941670; doi:10.3389/fgene.2023.1050696)
Supplement: Supplementary file 1 [file Table1.docx]

**Table 1** Characteristics of the datasets included in the analysis.

| **GEO ID** | **Platform** | **Citation** | **Region** | **Control** | **DCM** |
| --- | --- | --- | --- | --- | --- |
| GSE41177 | GPL570; Affymetrix Human Genome U133 Plus 2.0 Array | Ameling S, et al. Eur Heart J, 2013;34(9):666-75. PMID: 23100283 | Greifswald, Germany | 8 | 40 |
| GSE79962 | GPL6244; Affymetrix Human Gene 1.0 ST Array | Matkovich SJ, et al. Crit Care Med, 2017;45(3):407-414. PMID: 28067713 | St. Louis, USA | 11 | 9 |
| GSE3585 | GPL96; Affymetrix Human Genome U133A Array | Barth AS, et al. J Am Coll Cardiol 2006 Oct 17;48(8):1610-7. PMID: 17045896 | Heidelberg, Germany | 5 | 7 |

GEO: Gene Expression Omnibus; DCM: dilated cardiomyopathy.
