# Supplementary figures and images for: Integrated genomic analysis defines molecular subgroups in dilated cardiomyopathy and identifies novel biomarkers based on machine learning methods
Source: Front Genet. 2023 Feb 7;14:1050696. doi: 10.3389/fgene.2023.1050696 (PMC9941670; doi:10.3389/fgene.2023.1050696)

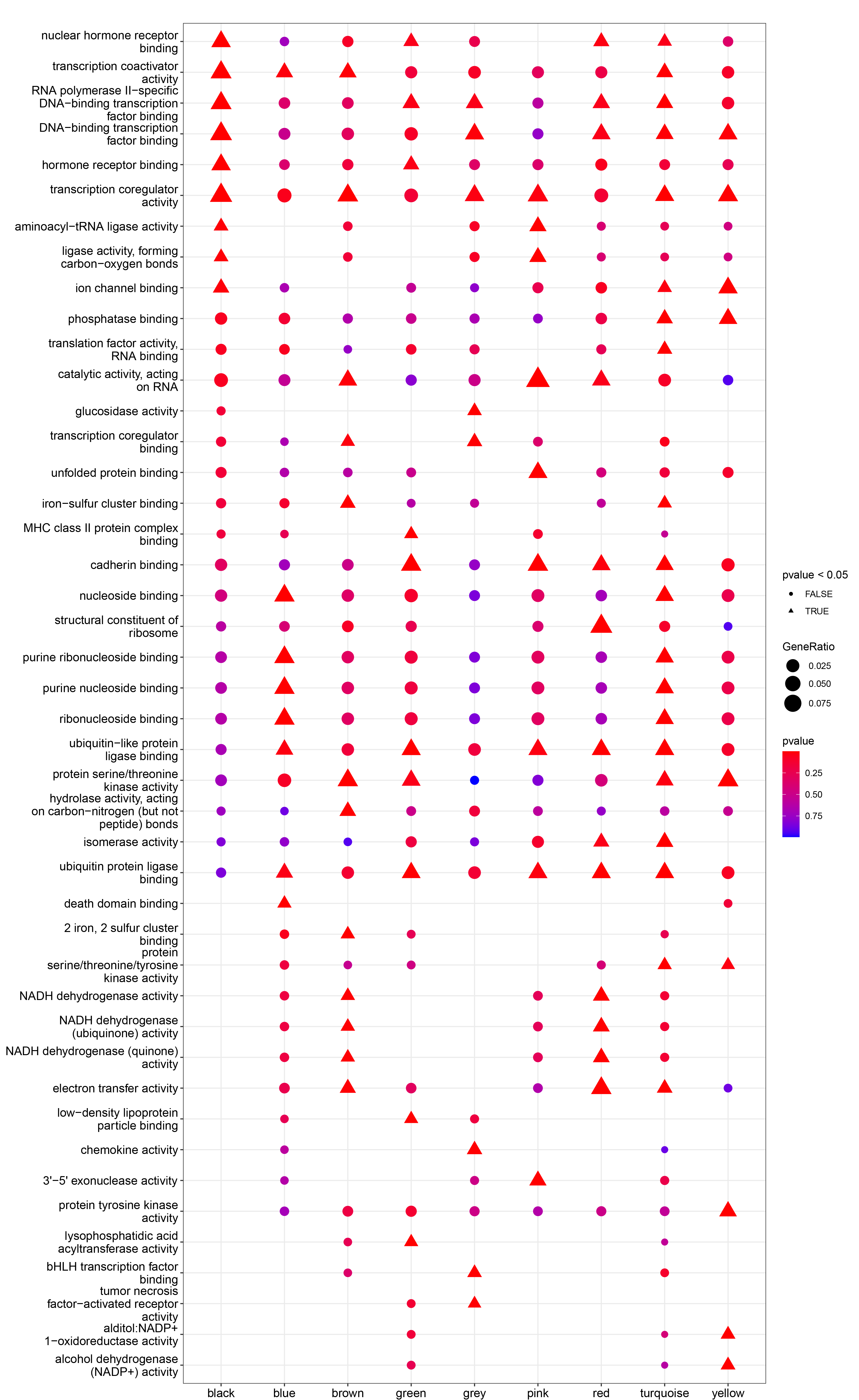

Supplement: Supplementary file 2 [file Image2.TIF]

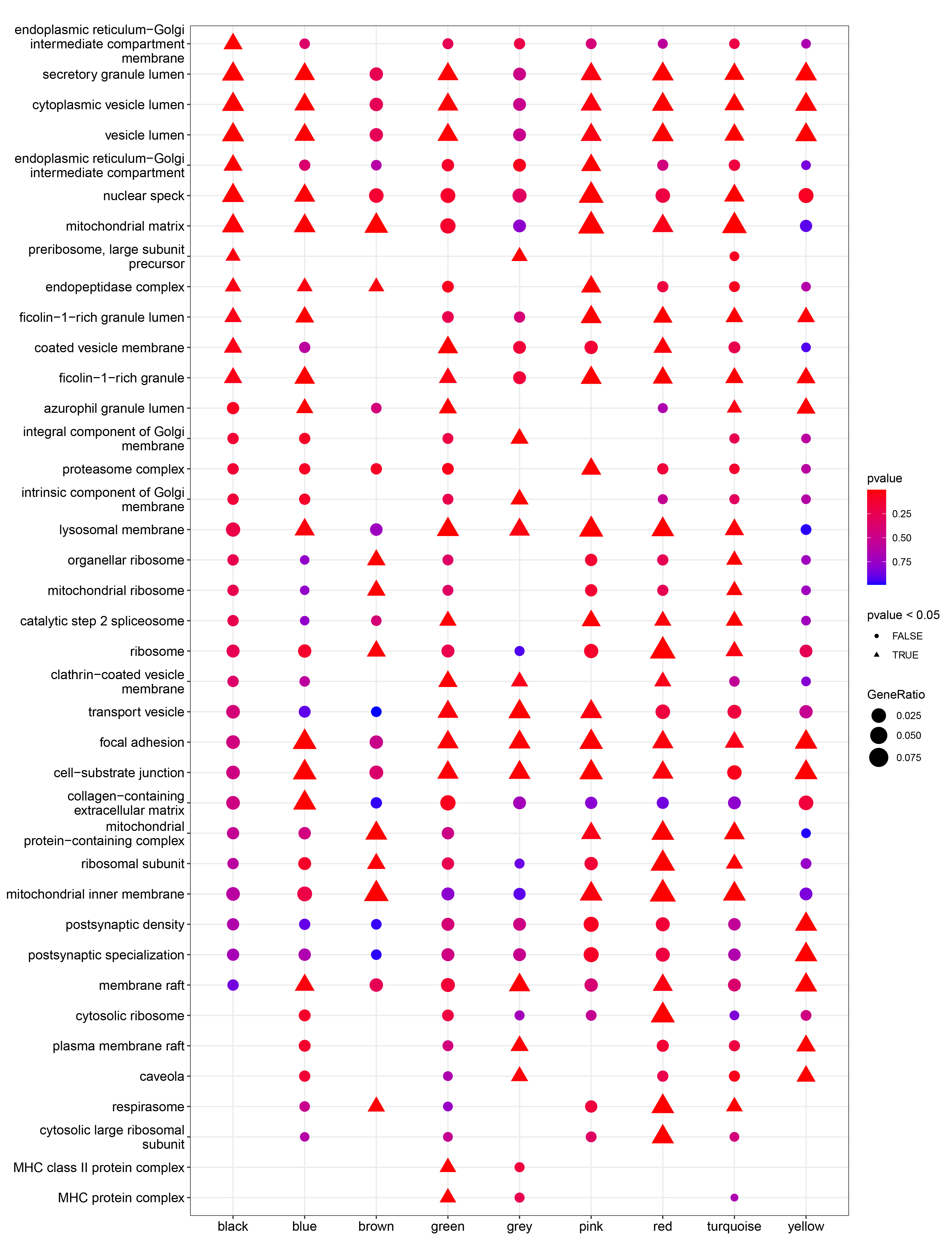

Supplement: Supplementary file 3 [file Image1.TIF]
