# Supplementary material for: Integrated genomic analysis defines molecular subgroups in dilated cardiomyopathy and identifies novel biomarkers based on machine learning methods
Source: Front Genet. 2023 Feb 7;14:1050696. doi: 10.3389/fgene.2023.1050696 (PMC9941670; doi:10.3389/fgene.2023.1050696)
Supplement: Supplementary file 4 [file Table2.docx]

**Table 2.** Analysis of variance for classification of subgroups, age, and their interactions.

|  | **Df** | **Sum square** | **Mean square** | **F value** | **Pr (>F)** |
| --- | --- | --- | --- | --- | --- |
| Subgroup | 2 | 411.4 | 205.6 | 6.03 | 0.006^∗∗^ |
| Age | 1 | 0.5 | 0.53 | 0.016 | 0.902 |
| Subgroup and age interaction  Residuals | 2  34 | 32.3  1159.8 | 16.13  34.11 | 0.473 | 0.63 |

Df: degree of freedom. Significant codes: “∗∗∗” 0.001, “∗∗” 0.01, “∗” 0.05.
