# Supplementary material for: Integrated genomic analysis defines molecular subgroups in dilated cardiomyopathy and identifies novel biomarkers based on machine learning methods
Source: Front Genet. 2023 Feb 7;14:1050696. doi: 10.3389/fgene.2023.1050696 (PMC9941670; doi:10.3389/fgene.2023.1050696)
Supplement: Supplementary file 5 [file Table5.docx]

**Table S5. All DEGs in subgroup 2.**

| **Gene names** | ***P* Value** | **Adjust *P* Value** |
| --- | --- | --- |
| PPP2R1A | <0.001 | <0.001 |
| DCTN1 | <0.001 | <0.001 |
| CALCOCO1 | <0.001 | <0.001 |
| MTUS1 | <0.001 | <0.001 |
| KAT7 | <0.001 | <0.001 |
| JTB | <0.001 | <0.001 |
| RERE | <0.001 | <0.001 |
| PGAP4 | <0.001 | <0.001 |
| ARHGEF9 | <0.001 | <0.001 |
| RWDD1 | <0.001 | <0.001 |
| CSRP2 | <0.001 | <0.001 |
| ALG5 | <0.001 | <0.001 |
| CAMK2B | <0.001 | <0.001 |
| IFT20 | <0.001 | <0.001 |
| COPS7A | <0.001 | <0.001 |
| NUCB1 | <0.001 | <0.001 |
| ROR1 | <0.001 | <0.001 |
| CYB5R1 | <0.001 | <0.001 |
| GCA | <0.001 | <0.001 |
| AKT1 | <0.001 | <0.001 |
| PLAU | <0.001 | <0.001 |
| MBD2 | <0.001 | <0.001 |
| LMO7 | <0.001 | <0.001 |
| BANF1 | <0.001 | <0.001 |
| TWIST1 | <0.001 | <0.001 |
| P2RY14 | <0.001 | <0.001 |
| PTPN14 | <0.001 | <0.001 |
| AKAP6 | <0.001 | <0.001 |
| ZNF266 | <0.001 | <0.001 |
| FXR2 | <0.001 | <0.001 |
| ANKRD17 | <0.001 | <0.001 |
| ZNF106 | <0.001 | <0.001 |
| USP13 | <0.001 | <0.001 |
| H2BC21 | <0.001 | <0.001 |
| PLA2G4A | <0.001 | <0.001 |
| DHTKD1 | <0.001 | <0.001 |
| PNPLA6 | <0.001 | <0.001 |
| NEU3 | <0.001 | <0.001 |
| HSP90AB1 | <0.001 | <0.001 |
| ILK | <0.001 | <0.001 |
| EFCAB11 | <0.001 | <0.001 |
| COPA | <0.001 | <0.001 |
| RCN2 | <0.001 | <0.001 |
| LITAF | <0.001 | <0.001 |
| ZKSCAN1 | <0.001 | <0.001 |
| GPI | <0.001 | <0.001 |
| ALOX5 | <0.001 | <0.001 |
| TCEAL4 | <0.001 | <0.001 |
| KPNA2 | <0.001 | <0.001 |
| ALPK3 | <0.001 | <0.001 |
| PGC | <0.001 | <0.001 |
| KPNA6 | <0.001 | <0.001 |
| SAT1 | <0.001 | <0.001 |
| TUBA1A | <0.001 | <0.001 |
| MPV17 | <0.001 | <0.001 |
| RBBP6 | <0.001 | <0.001 |
| WBP2 | <0.001 | <0.001 |
| CSNK2B | <0.001 | <0.001 |
| FMO4 | <0.001 | <0.001 |
| EEF1B2 | <0.001 | <0.001 |
| CAPNS1 | <0.001 | <0.001 |
| ATXN10 | <0.001 | <0.001 |
| HSPB11 | <0.001 | <0.001 |
| PIP4K2B | <0.001 | <0.001 |
| PRKACA | <0.001 | <0.001 |
| IL13RA1 | <0.001 | <0.001 |
| LARGE1 | <0.001 | <0.001 |
| PDLIM1 | <0.001 | <0.001 |
| RANBP2 | <0.001 | <0.001 |
| MBD4 | <0.001 | <0.001 |
| KANK1 | <0.001 | <0.001 |
| SEPHS2 | <0.001 | <0.001 |
| DET1 | <0.001 | <0.001 |
| MAP3K8 | <0.001 | <0.001 |
| ARHGEF12 | <0.001 | <0.001 |
| CDK16 | <0.001 | <0.001 |
| DUSP8 | <0.001 | <0.001 |
| ARL6IP1 | <0.001 | <0.001 |
| MLF2 | <0.001 | <0.001 |
| DUT | <0.001 | <0.001 |
| MED7 | <0.001 | <0.001 |
| ECI2 | <0.001 | <0.001 |
| HSPA5 | <0.001 | <0.001 |
| DVL3 | <0.001 | <0.001 |
| POLDIP2 | <0.001 | <0.001 |
| WDFY3 | <0.001 | <0.001 |
| MFN2 | <0.001 | <0.001 |
| MRFAP1L1 | <0.001 | <0.001 |
| FBXL5 | <0.001 | <0.001 |
| GYPC | <0.001 | <0.001 |
| SSBP3 | <0.001 | <0.001 |
| XPO7 | <0.001 | <0.001 |
| SYNPO2L | <0.001 | <0.001 |
| HSPB1 | <0.001 | <0.001 |
| ANK2 | <0.001 | <0.001 |
| SCMH1 | <0.001 | <0.001 |
| GYS1 | <0.001 | <0.001 |
| TCFL5 | <0.001 | <0.001 |
| GCLM | <0.001 | <0.001 |
| RNF114 | <0.001 | <0.001 |
| ATP1A3 | <0.001 | <0.001 |
| GRINA | <0.001 | <0.001 |
| RBM5 | <0.001 | <0.001 |
| MYL9 | <0.001 | <0.001 |
| POLR2H | <0.001 | <0.001 |
| RAP2B | <0.001 | <0.001 |
| HIGD2A | <0.001 | <0.001 |
| NME4 | <0.001 | <0.001 |
| TMEM14A | <0.001 | <0.001 |
| FTO | <0.001 | <0.001 |
| AKR7A2 | <0.001 | <0.001 |
| NAT10 | <0.001 | <0.001 |
| ACTA1 | <0.001 | <0.001 |
| MSR1 | <0.001 | <0.001 |
| VAMP2 | <0.001 | <0.001 |
| SPTSSA | <0.001 | <0.001 |
| PYGB | <0.001 | <0.001 |
| RETREG2 | <0.001 | <0.001 |
| MRPS22 | <0.001 | <0.001 |
| YY1 | <0.001 | <0.001 |
| USP11 | <0.001 | <0.001 |
| AKR1C1 | <0.001 | <0.001 |
| PNPLA2 | <0.001 | <0.001 |
| ABHD3 | <0.001 | <0.001 |
| RTL8C | <0.001 | <0.001 |
| SMYD2 | <0.001 | <0.001 |
| BIRC2 | <0.001 | <0.001 |
| MNDA | <0.001 | <0.001 |
| FLOT2 | <0.001 | <0.001 |
| NAPA | <0.001 | <0.001 |
| PPP2CB | <0.001 | <0.001 |
| LY96 | <0.001 | <0.001 |
| ATP6V0E1 | <0.001 | <0.001 |
| TMCO1 | <0.001 | <0.001 |
| FGF1 | <0.001 | <0.001 |
| PDCD2 | <0.001 | <0.001 |
| ATP2B4 | <0.001 | <0.001 |
| CCDC90B | <0.001 | <0.001 |
| MED4 | <0.001 | <0.001 |
| UTP18 | <0.001 | <0.001 |
| SGMS1 | <0.001 | <0.001 |
| TRMT5 | <0.001 | <0.001 |
| AKAP13 | <0.001 | <0.001 |
| UBE2E1 | <0.001 | 0.001 |
| DHRS11 | <0.001 | 0.001 |
| PNRC1 | <0.001 | 0.001 |
| ALDOC | <0.001 | 0.001 |
| VDAC2 | <0.001 | 0.001 |
| MRPL20 | <0.001 | 0.001 |
| VPS45 | <0.001 | 0.001 |
| PPIC | <0.001 | 0.001 |
| ACO2 | <0.001 | 0.001 |
| NFIC | <0.001 | 0.001 |
| JAK1 | <0.001 | 0.001 |
| LCP2 | <0.001 | 0.001 |
| OXA1L | <0.001 | 0.001 |
| KCMF1 | <0.001 | 0.001 |
| TMEM165 | <0.001 | 0.001 |
| RAD23A | <0.001 | 0.001 |
| SH3YL1 | <0.001 | 0.001 |
| LMCD1 | <0.001 | 0.001 |
| FGL2 | <0.001 | 0.001 |
| HSPBAP1 | <0.001 | 0.001 |
| RBL2 | <0.001 | 0.001 |
| COX6B1 | <0.001 | 0.001 |
| EEF1A2 | <0.001 | 0.001 |
| HMGN5 | <0.001 | 0.001 |
| BTRC | <0.001 | 0.001 |
| PSMD11 | <0.001 | 0.001 |
| GULP1 | <0.001 | 0.001 |
| SUPT6H | <0.001 | 0.001 |
| DNAJC11 | <0.001 | 0.001 |
| TCF3 | <0.001 | 0.001 |
| PABPC4 | <0.001 | 0.001 |
| GGCX | <0.001 | 0.001 |
| ECH1 | <0.001 | 0.001 |
| CYP2J2 | <0.001 | 0.001 |
| CNOT7 | <0.001 | 0.001 |
| MALT1 | <0.001 | 0.001 |
| NUP54 | <0.001 | 0.001 |
| PSMD7 | <0.001 | 0.001 |
| GTF3C1 | <0.001 | 0.001 |
| BCL6 | <0.001 | 0.001 |
| DHFR | <0.001 | 0.001 |
| STRAP | <0.001 | 0.001 |
| GABRE | <0.001 | 0.001 |
| ARCN1 | <0.001 | 0.001 |
| DDB1 | <0.001 | 0.001 |
| ADCY7 | <0.001 | 0.001 |
| NQO1 | <0.001 | 0.001 |
| SMARCB1 | <0.001 | 0.001 |
| TGM2 | <0.001 | 0.001 |
| ATP6V1B2 | <0.001 | 0.001 |
| HPF1 | <0.001 | 0.001 |
| TOMM34 | <0.001 | 0.001 |
| CREBL2 | <0.001 | 0.001 |
| EXOC6B | <0.001 | 0.001 |
| IRX5 | <0.001 | 0.001 |
| NADK | <0.001 | 0.001 |
| TFDP2 | <0.001 | 0.001 |
| APIP | <0.001 | 0.001 |
| TPCN1 | <0.001 | 0.001 |
| ERC1 | <0.001 | 0.001 |
| PRPF40A | <0.001 | 0.001 |
| INSIG1 | <0.001 | 0.001 |
| NIT1 | <0.001 | 0.001 |
| UPF3A | <0.001 | 0.001 |
| EHD3 | <0.001 | 0.001 |
| PAPOLA | <0.001 | 0.001 |
| HOMER2 | <0.001 | 0.001 |
| RYK | <0.001 | 0.001 |
| KIFBP | <0.001 | 0.001 |
| NCDN | <0.001 | 0.001 |
| PSPC1 | <0.001 | 0.001 |
| ATP6AP1 | <0.001 | 0.001 |
| CSGALNACT2 | <0.001 | 0.001 |
| TRIM5 | <0.001 | 0.001 |
| APLP2 | <0.001 | 0.001 |
| MAOB | <0.001 | 0.001 |
| NT5E | <0.001 | 0.001 |
| EBNA1BP2 | <0.001 | 0.001 |
| SERP1 | <0.001 | 0.001 |
| SNRNP200 | <0.001 | 0.001 |
| RELA | <0.001 | 0.001 |
| SNX1 | <0.001 | 0.001 |
| RABEP2 | <0.001 | 0.001 |
| JAM3 | <0.001 | 0.001 |
| TNS1 | <0.001 | 0.001 |
| TMEM131L | <0.001 | 0.001 |
| IFT57 | <0.001 | 0.001 |
| TMEM222 | <0.001 | 0.001 |
| TNFSF10 | <0.001 | 0.001 |
| PDK2 | <0.001 | 0.001 |
| TMUB2 | <0.001 | 0.001 |
| CRYAB | <0.001 | 0.001 |
| TMEM109 | <0.001 | 0.001 |
| BMERB1 | <0.001 | 0.001 |
| CASQ1 | <0.001 | 0.001 |
| HNMT | <0.001 | 0.001 |
| GRIP2 | <0.001 | 0.001 |
| TOMM40 | <0.001 | 0.001 |
| SEMA5A | <0.001 | 0.001 |
| CLN5 | <0.001 | 0.001 |
| METAP2 | <0.001 | 0.001 |
| HSPA14 | <0.001 | 0.001 |
| MAP4K5 | <0.001 | 0.001 |
| FGFR2 | <0.001 | 0.001 |
| MAP2K7 | <0.001 | 0.001 |
| DES | <0.001 | 0.001 |
| FAM189A2 | <0.001 | 0.001 |
| MYOM1 | <0.001 | 0.001 |
| TIMM10B | <0.001 | 0.001 |
| LBR | <0.001 | 0.001 |
| PRMT1 | <0.001 | 0.001 |
| MTIF2 | <0.001 | 0.001 |
| ZMYM3 | <0.001 | 0.001 |
| IPO13 | <0.001 | 0.001 |
| SLC8A1 | <0.001 | 0.001 |
| DDX24 | <0.001 | 0.001 |
| ALAS1 | <0.001 | 0.001 |
| CLUH | <0.001 | 0.001 |
| NSDHL | <0.001 | 0.001 |
| FBXO21 | <0.001 | 0.001 |
| VGLL4 | <0.001 | 0.001 |
| MAZ | <0.001 | 0.001 |
| MRPS18B | <0.001 | 0.001 |
| PRKAG1 | <0.001 | 0.001 |
| SRGN | <0.001 | 0.001 |
| TTC31 | <0.001 | 0.001 |
| ZNF146 | <0.001 | 0.001 |
| RBM23 | <0.001 | 0.001 |
| CDV3 | <0.001 | 0.001 |
| FXYD1 | <0.001 | 0.001 |
| TIMM17A | <0.001 | 0.001 |
| WNK1 | <0.001 | 0.001 |
| TM2D1 | <0.001 | 0.001 |
| EVI2A | <0.001 | 0.001 |
| TERF2 | <0.001 | 0.001 |
| PRKD1 | <0.001 | 0.001 |
| CMTM6 | <0.001 | 0.001 |
| CCNG2 | <0.001 | 0.001 |
| GOT1 | <0.001 | 0.001 |
| OLFML2A | <0.001 | 0.001 |
| ZFPL1 | <0.001 | 0.001 |
| TRIM28 | <0.001 | 0.001 |
| MS4A6A | <0.001 | 0.001 |
| DHPS | <0.001 | 0.001 |
| NTAN1 | <0.001 | 0.001 |
| IFI16 | <0.001 | 0.001 |
| GAB1 | <0.001 | 0.001 |
| IFFO1 | <0.001 | 0.001 |
| BSG | <0.001 | 0.001 |
| SELENOP | <0.001 | 0.001 |
| ARHGEF17 | <0.001 | 0.001 |
| DPM1 | <0.001 | 0.001 |
| NXF1 | <0.001 | 0.001 |
| TERF2IP | <0.001 | 0.002 |
| OSTF1 | <0.001 | 0.002 |
| ARF1 | <0.001 | 0.002 |
| RER1 | <0.001 | 0.002 |
| SAR1B | <0.001 | 0.002 |
| RPIA | <0.001 | 0.002 |
| UBA2 | <0.001 | 0.002 |
| TMA16 | <0.001 | 0.002 |
| TCP1 | <0.001 | 0.002 |
| SMAD1 | <0.001 | 0.002 |
| ACACB | <0.001 | 0.002 |
| PKM | <0.001 | 0.002 |
| TWF1 | <0.001 | 0.002 |
| RACK1 | <0.001 | 0.002 |
| ANXA6 | <0.001 | 0.002 |
| RRAGC | <0.001 | 0.002 |
| KLHL2 | <0.001 | 0.002 |
| PPP2R2A | <0.001 | 0.002 |
| GSAP | <0.001 | 0.002 |
| CENPB | <0.001 | 0.002 |
| CRLF3 | <0.001 | 0.002 |
| AK2 | <0.001 | 0.002 |
| TFG | <0.001 | 0.002 |
| PDE6D | <0.001 | 0.002 |
| DNAJC8 | <0.001 | 0.002 |
| SMC3 | <0.001 | 0.002 |
| NET1 | <0.001 | 0.002 |
| IL10RA | <0.001 | 0.002 |
| NMT2 | <0.001 | 0.002 |
| AURKA | <0.001 | 0.002 |
| AP2M1 | <0.001 | 0.002 |
| SCN5A | <0.001 | 0.002 |
| BCL7B | <0.001 | 0.002 |
| UQCRFS1 | <0.001 | 0.002 |
| EMP1 | <0.001 | 0.002 |
| ZNF384 | <0.001 | 0.002 |
| NR1H2 | <0.001 | 0.002 |
| CKAP5 | <0.001 | 0.002 |
| AKR1C3 | <0.001 | 0.002 |
| CHSY1 | <0.001 | 0.002 |
| STK3 | <0.001 | 0.002 |
| MIIP | <0.001 | 0.002 |
| GSTM1 | <0.001 | 0.002 |
| USP24 | <0.001 | 0.002 |
| NNT | <0.001 | 0.002 |
| FBXO5 | <0.001 | 0.002 |
| MPP1 | <0.001 | 0.002 |
| TAF11 | <0.001 | 0.002 |
| C2orf42 | <0.001 | 0.002 |
| TMEM9B | <0.001 | 0.002 |
| ARRB1 | <0.001 | 0.002 |
| RPL3L | <0.001 | 0.002 |
| ADAP2 | <0.001 | 0.002 |
| PFKFB3 | <0.001 | 0.002 |
| XPA | <0.001 | 0.002 |
| PPM1G | <0.001 | 0.002 |
| SMARCD1 | <0.001 | 0.002 |
| DYRK4 | <0.001 | 0.002 |
| NES | <0.001 | 0.002 |
| SARAF | <0.001 | 0.002 |
| METTL5 | <0.001 | 0.002 |
| HIP1 | <0.001 | 0.002 |
| DDX1 | <0.001 | 0.002 |
| MFSD1 | <0.001 | 0.002 |
| N4BP2L1 | <0.001 | 0.002 |
| FBXO42 | <0.001 | 0.002 |
| APH1A | <0.001 | 0.002 |
| NFYB | <0.001 | 0.002 |
| PFDN1 | <0.001 | 0.002 |
| PLOD2 | <0.001 | 0.002 |
| MPP2 | <0.001 | 0.002 |
| PTPN2 | <0.001 | 0.002 |
| LDAH | <0.001 | 0.002 |
| CLN3 | <0.001 | 0.002 |
| LAMP2 | <0.001 | 0.002 |
| C19orf53 | <0.001 | 0.002 |
| DFFA | <0.001 | 0.002 |
| MED14 | <0.001 | 0.003 |
| CEBPZ | <0.001 | 0.003 |
| CAPN2 | <0.001 | 0.003 |
| KCND3 | <0.001 | 0.003 |
| EIF4H | <0.001 | 0.003 |
| ABTB2 | <0.001 | 0.003 |
| SNAI2 | <0.001 | 0.003 |
| CAPZA1 | <0.001 | 0.003 |
| PTPN21 | <0.001 | 0.003 |
| ACP1 | <0.001 | 0.003 |
| CCT8 | <0.001 | 0.003 |
| ZFAND3 | <0.001 | 0.003 |
| AAMDC | <0.001 | 0.003 |
| RPAP3 | <0.001 | 0.003 |
| MAP2K3 | <0.001 | 0.003 |
| FAXDC2 | <0.001 | 0.003 |
| NCKAP1 | <0.001 | 0.003 |
| CSDC2 | <0.001 | 0.003 |
| BLNK | <0.001 | 0.003 |
| ERO1A | <0.001 | 0.003 |
| APOOL | <0.001 | 0.003 |
| DMXL1 | <0.001 | 0.003 |
| PKD1 | <0.001 | 0.003 |
| INVS | <0.001 | 0.003 |
| ARFRP1 | <0.001 | 0.003 |
| FRG1 | <0.001 | 0.003 |
| AREG | <0.001 | 0.003 |
| PRLH | <0.001 | 0.003 |
| MGST3 | <0.001 | 0.003 |
| NAA35 | <0.001 | 0.003 |
| KLF10 | <0.001 | 0.003 |
| NBAS | <0.001 | 0.003 |
| SAP18 | <0.001 | 0.003 |
| MTHFD2 | <0.001 | 0.003 |
| ERGIC3 | <0.001 | 0.003 |
| GMPR2 | <0.001 | 0.003 |
| SYNE1 | <0.001 | 0.003 |
| SRSF4 | <0.001 | 0.003 |
| ACIN1 | <0.001 | 0.003 |
| FHOD3 | <0.001 | 0.003 |
| EPB41L3 | <0.001 | 0.003 |
| PSG6 | <0.001 | 0.003 |
| ATP5MC2 | <0.001 | 0.003 |
| BABAM2 | <0.001 | 0.003 |
| ARFGAP2 | <0.001 | 0.003 |
| ELL3 | <0.001 | 0.003 |
| ISG15 | <0.001 | 0.003 |
| MTMR14 | <0.001 | 0.003 |
| DMPK | <0.001 | 0.003 |
| QRSL1 | <0.001 | 0.003 |
| SNRPA1 | <0.001 | 0.003 |
| CRIP2 | <0.001 | 0.003 |
| PARP2 | <0.001 | 0.003 |
| KALRN | <0.001 | 0.003 |
| PLOD3 | <0.001 | 0.003 |
| SYNC | <0.001 | 0.003 |
| RRP15 | <0.001 | 0.003 |
| CAP1 | <0.001 | 0.003 |
| PPA1 | <0.001 | 0.003 |
| TXN | <0.001 | 0.003 |
| KLF4 | <0.001 | 0.003 |
| XPO6 | <0.001 | 0.004 |
| BBLN | <0.001 | 0.004 |
| GADD45A | <0.001 | 0.004 |
| GATA4 | <0.001 | 0.004 |
| FADS1 | <0.001 | 0.004 |
| RPAP1 | <0.001 | 0.004 |
| ABR | <0.001 | 0.004 |
| FAIM | <0.001 | 0.004 |
| MPHOSPH10 | <0.001 | 0.004 |
| GPN3 | <0.001 | 0.004 |
| DENND5A | <0.001 | 0.004 |
| CSF2RB | <0.001 | 0.004 |
| TMEM45A | <0.001 | 0.004 |
| SSH3 | <0.001 | 0.004 |
| SMTN | <0.001 | 0.004 |
| SETX | <0.001 | 0.004 |
| IGHMBP2 | <0.001 | 0.004 |
| NDRG4 | <0.001 | 0.004 |
| APAF1 | <0.001 | 0.004 |
| DOP1A | <0.001 | 0.004 |
| C19orf54 | <0.001 | 0.004 |
| HDAC6 | <0.001 | 0.004 |
| SQSTM1 | <0.001 | 0.004 |
| SRPRA | <0.001 | 0.004 |
| XPNPEP1 | <0.001 | 0.004 |
| R3HDM2 | <0.001 | 0.004 |
| SEPTIN8 | <0.001 | 0.004 |
| ATP6V1H | <0.001 | 0.004 |
| CLK3 | <0.001 | 0.004 |
| NRDC | <0.001 | 0.004 |
| IRAK1 | <0.001 | 0.004 |
| SPATA2L | <0.001 | 0.004 |
| LYPLA2 | <0.001 | 0.004 |
| TSPAN9 | <0.001 | 0.004 |
| MIS12 | <0.001 | 0.004 |
| SAMSN1 | <0.001 | 0.004 |
| VCP | <0.001 | 0.004 |
| AP3B1 | <0.001 | 0.004 |
| HERC1 | <0.001 | 0.004 |
| ABCF1 | <0.001 | 0.004 |
| TEFM | <0.001 | 0.004 |
| INTS9 | <0.001 | 0.004 |
| IDH3G | <0.001 | 0.004 |
| PRDX4 | <0.001 | 0.004 |
| NSFL1C | <0.001 | 0.004 |
| ARPC2 | <0.001 | 0.004 |
| TBL1XR1 | <0.001 | 0.004 |
| TMED2 | <0.001 | 0.004 |
| UQCR10 | <0.001 | 0.004 |
| TUBB | <0.001 | 0.004 |
| PPP1CC | <0.001 | 0.004 |
| FABP4 | <0.001 | 0.004 |
| RHOA | <0.001 | 0.004 |
| SLC38A6 | <0.001 | 0.004 |
| ACAD8 | <0.001 | 0.004 |
| KLHL24 | <0.001 | 0.004 |
| CPNE3 | <0.001 | 0.004 |
| GAS7 | <0.001 | 0.004 |
| MAT2A | <0.001 | 0.004 |
| CADM1 | <0.001 | 0.004 |
| ZCCHC8 | <0.001 | 0.004 |
| ATG14 | <0.001 | 0.004 |
| UBA1 | <0.001 | 0.005 |
| PSEN1 | <0.001 | 0.005 |
| HMBOX1 | <0.001 | 0.005 |
| GSE1 | <0.001 | 0.005 |
| DPYD | <0.001 | 0.005 |
| PSMC3IP | <0.001 | 0.005 |
| HARS2 | <0.001 | 0.005 |
| CORO1C | <0.001 | 0.005 |
| ATP1B3 | <0.001 | 0.005 |
| ERLIN1 | <0.001 | 0.005 |
| MKRN1 | <0.001 | 0.005 |
| TAFAZZIN | <0.001 | 0.005 |
| CHD4 | <0.001 | 0.005 |
| ELOVL5 | <0.001 | 0.005 |
| FAM3A | <0.001 | 0.005 |
| ABITRAM | <0.001 | 0.005 |
| ZFPM2 | <0.001 | 0.005 |
| JAK2 | <0.001 | 0.005 |
| ARHGEF11 | <0.001 | 0.005 |
| PROCR | <0.001 | 0.005 |
| RNF41 | <0.001 | 0.005 |
| CASZ1 | <0.001 | 0.005 |
| TIMM8A | <0.001 | 0.005 |
| N4BP1 | <0.001 | 0.005 |
| FAS | <0.001 | 0.005 |
| TRIP10 | <0.001 | 0.005 |
| SLC35E3 | <0.001 | 0.005 |
| NDUFA13 | <0.001 | 0.005 |
| CYFIP1 | <0.001 | 0.005 |
| NCAM1 | <0.001 | 0.005 |
| TNFRSF14 | <0.001 | 0.005 |
| ALDH2 | <0.001 | 0.005 |
| NFKB1 | <0.001 | 0.005 |
| APOBEC3C | <0.001 | 0.005 |
| TANK | <0.001 | 0.005 |
| SLC35E1 | <0.001 | 0.005 |
| PRDX3 | <0.001 | 0.005 |
| EHD1 | <0.001 | 0.005 |
| PLXNC1 | <0.001 | 0.005 |
| METTL16 | <0.001 | 0.005 |
| SGCB | <0.001 | 0.005 |
| SRPRB | <0.001 | 0.005 |
| BBS10 | <0.001 | 0.005 |
| PPP1R15A | <0.001 | 0.005 |
| AAK1 | <0.001 | 0.005 |
| PDSS2 | <0.001 | 0.005 |
| VAMP7 | <0.001 | 0.005 |
| CTSD | <0.001 | 0.005 |
| SRM | <0.001 | 0.005 |
| PPM1D | <0.001 | 0.005 |
| NGLY1 | <0.001 | 0.005 |
| CHMP2B | <0.001 | 0.005 |
| ATXN2 | <0.001 | 0.005 |
| CEBPB | <0.001 | 0.005 |
| C1orf174 | <0.001 | 0.005 |
| IFT46 | <0.001 | 0.005 |
| TUBG1 | <0.001 | 0.005 |
| CAMLG | <0.001 | 0.005 |
| VPS39 | <0.001 | 0.005 |
| FKTN | <0.001 | 0.005 |
| MLYCD | <0.001 | 0.005 |
| ZBTB5 | <0.001 | 0.005 |
| PAQR3 | <0.001 | 0.005 |
| LAMB2 | <0.001 | 0.005 |
| NECTIN3 | <0.001 | 0.005 |
| AGTR1 | <0.001 | 0.006 |
| CCNI | <0.001 | 0.006 |
| OARD1 | <0.001 | 0.006 |
| C7orf25 | <0.001 | 0.006 |
| MED24 | <0.001 | 0.006 |
| ARL6IP5 | <0.001 | 0.006 |
| NPRL3 | <0.001 | 0.006 |
| SNRPB | <0.001 | 0.006 |
| ZBTB43 | <0.001 | 0.006 |
| MRPS7 | <0.001 | 0.006 |
| PTPRC | <0.001 | 0.006 |
| SHLD2 | <0.001 | 0.006 |
| ARHGAP1 | <0.001 | 0.006 |
| MCCC2 | <0.001 | 0.006 |
| TBL2 | <0.001 | 0.006 |
| ORC3 | <0.001 | 0.006 |
| THOC7 | <0.001 | 0.006 |
| TSC1 | <0.001 | 0.006 |
| PTP4A2 | <0.001 | 0.006 |
| MCCC1 | <0.001 | 0.006 |
| AKR1C2 | <0.001 | 0.006 |
| RPL26L1 | <0.001 | 0.006 |
| NKRF | <0.001 | 0.006 |
| PIK3R2 | <0.001 | 0.006 |
| ID4 | <0.001 | 0.006 |
| CALCOCO2 | 0.001 | 0.006 |
| SSX2IP | 0.001 | 0.006 |
| ATG13 | 0.001 | 0.006 |
| ACTR1A | 0.001 | 0.006 |
| NEDD4L | 0.001 | 0.006 |
| AIFM1 | 0.001 | 0.006 |
| RFK | 0.001 | 0.006 |
| PQBP1 | 0.001 | 0.006 |
| CCNC | 0.001 | 0.006 |
| MCM6 | 0.001 | 0.006 |
| PRPS2 | 0.001 | 0.006 |
| CXCR4 | 0.001 | 0.006 |
| DPF3 | 0.001 | 0.006 |
| FAM168B | 0.001 | 0.006 |
| ASB8 | 0.001 | 0.006 |
| GRK5 | 0.001 | 0.006 |
| TUBD1 | 0.001 | 0.006 |
| DDX49 | 0.001 | 0.007 |
| PIGK | 0.001 | 0.007 |
| APBB3 | 0.001 | 0.007 |
| VPS72 | 0.001 | 0.007 |
| BRD2 | 0.001 | 0.007 |
| DESI1 | 0.001 | 0.007 |
| ZNF131 | 0.001 | 0.007 |
| SPTAN1 | 0.001 | 0.007 |
| HBP1 | 0.001 | 0.007 |
| BCCIP | 0.001 | 0.007 |
| MCTS1 | 0.001 | 0.007 |
| PPWD1 | 0.001 | 0.007 |
| BAG6 | 0.001 | 0.007 |
| PDLIM2 | 0.001 | 0.007 |
| MYCBP2 | 0.001 | 0.007 |
| POPDC2 | 0.001 | 0.007 |
| ACLY | 0.001 | 0.007 |
| NUCB2 | 0.001 | 0.007 |
| PHC2 | 0.001 | 0.007 |
| EXT1 | 0.001 | 0.007 |
| TRMT1L | 0.001 | 0.007 |
| MS4A4A | 0.001 | 0.007 |
| BTBD1 | 0.001 | 0.007 |
| EXT2 | 0.001 | 0.007 |
| BMP2K | 0.001 | 0.007 |
| PRRC2A | 0.001 | 0.007 |
| MRPS18A | 0.001 | 0.007 |
| MAGI1 | 0.001 | 0.007 |
| EPS8 | 0.001 | 0.007 |
| BPNT2 | 0.001 | 0.007 |
| MAP1LC3B | 0.001 | 0.007 |
| TPI1 | 0.001 | 0.007 |
| TMX1 | 0.001 | 0.007 |
| MTERF3 | 0.001 | 0.007 |
| SIRPA | 0.001 | 0.007 |
| EIF2B5 | 0.001 | 0.007 |
| WRAP53 | 0.001 | 0.007 |
| MPRIP | 0.001 | 0.007 |
| NOL7 | 0.001 | 0.007 |
| PKP2 | 0.001 | 0.007 |
| H1-0 | 0.001 | 0.007 |
| ECT2 | 0.001 | 0.007 |
| PPIF | 0.001 | 0.007 |
| CD74 | 0.001 | 0.007 |
| ANKRD6 | 0.001 | 0.007 |
| KLHDC3 | 0.001 | 0.007 |
| PPME1 | 0.001 | 0.007 |
| SUB1 | 0.001 | 0.008 |
| BLVRA | 0.001 | 0.008 |
| FNTA | 0.001 | 0.008 |
| S100A1 | 0.001 | 0.008 |
| PKMYT1 | 0.001 | 0.008 |
| GARRE1 | 0.001 | 0.008 |
| PMS1 | 0.001 | 0.008 |
| C1QL1 | 0.001 | 0.008 |
| PRDX6 | 0.001 | 0.008 |
| UBTF | 0.001 | 0.008 |
| FBXL7 | 0.001 | 0.008 |
| ULK2 | 0.001 | 0.008 |
| WASHC3 | 0.001 | 0.008 |
| SP3 | 0.001 | 0.008 |
| VCL | 0.001 | 0.008 |
| TBRG4 | 0.001 | 0.008 |
| MAN2C1 | 0.001 | 0.008 |
| CLOCK | 0.001 | 0.008 |
| WEE1 | 0.001 | 0.008 |
| CAPZA2 | 0.001 | 0.008 |
| SARS1 | 0.001 | 0.008 |
| FHIT | 0.001 | 0.008 |
| PCCB | 0.001 | 0.008 |
| PKN1 | 0.001 | 0.008 |
| PHLDA2 | 0.001 | 0.008 |
| SLC9A2 | 0.001 | 0.008 |
| MTO1 | 0.001 | 0.008 |
| MRPL19 | 0.001 | 0.008 |
| BCKDHA | 0.001 | 0.008 |
| GORASP1 | 0.001 | 0.008 |
| NOL11 | 0.001 | 0.008 |
| DENR | 0.001 | 0.008 |
| LZTFL1 | 0.001 | 0.008 |
| ADIPOR1 | 0.001 | 0.008 |
| ABCF3 | 0.001 | 0.008 |
| TMEM208 | 0.001 | 0.008 |
| PPP1R3D | 0.001 | 0.008 |
| NRAP | 0.001 | 0.008 |
| TAPBP | 0.001 | 0.008 |
| DEPTOR | 0.001 | 0.008 |
| GDI1 | 0.001 | 0.008 |
| GOLGA7 | 0.001 | 0.008 |
| DIRAS3 | 0.001 | 0.008 |
| TMEM50B | 0.001 | 0.008 |
| IRF3 | 0.001 | 0.008 |
| TDRD7 | 0.001 | 0.008 |
| JUP | 0.001 | 0.008 |
| FAF1 | 0.001 | 0.008 |
| ISCA1 | 0.001 | 0.008 |
| DUSP22 | 0.001 | 0.009 |
| ZFP36L1 | 0.001 | 0.009 |
| RBM42 | 0.001 | 0.009 |
| MED6 | 0.001 | 0.009 |
| LGALS3BP | 0.001 | 0.009 |
| GGH | 0.001 | 0.009 |
| GPS1 | 0.001 | 0.009 |
| DOCK1 | 0.001 | 0.009 |
| GATAD1 | 0.001 | 0.009 |
| MGA | 0.001 | 0.009 |
| CCSER2 | 0.001 | 0.009 |
| SCML1 | 0.001 | 0.009 |
| ZNF226 | 0.001 | 0.009 |
| VEGFB | 0.001 | 0.009 |
| GCH1 | 0.001 | 0.009 |
| NIPSNAP1 | 0.001 | 0.009 |
| SNRNP70 | 0.001 | 0.009 |
| GLRX3 | 0.001 | 0.009 |
| PI4K2A | 0.001 | 0.009 |
| TNPO3 | 0.001 | 0.009 |
| TMEM223 | 0.001 | 0.009 |
| ZC3H15 | 0.001 | 0.009 |
| YARS2 | 0.001 | 0.009 |
| CXCL1 | 0.001 | 0.009 |
| IGSF6 | 0.001 | 0.009 |
| HSPB7 | 0.001 | 0.009 |
| SLIRP | 0.001 | 0.009 |
| PPP1R12B | 0.001 | 0.009 |
| LHFPL6 | 0.001 | 0.009 |
| THTPA | 0.001 | 0.009 |
| DNAJC2 | 0.001 | 0.01 |
| TBPL1 | 0.001 | 0.01 |
| TLE4 | 0.001 | 0.01 |
| DHRS9 | 0.001 | 0.01 |
| TBC1D8 | 0.001 | 0.01 |
| PCTP | 0.001 | 0.01 |
| MGAT2 | 0.001 | 0.01 |
| RAD50 | 0.001 | 0.01 |
| PFKL | 0.001 | 0.01 |
| RAB28 | 0.001 | 0.01 |
| ATP9B | 0.001 | 0.01 |
| ATP6V0C | 0.001 | 0.01 |
| B4GALT5 | 0.001 | 0.01 |
| PGGT1B | 0.001 | 0.01 |
| CETN2 | 0.001 | 0.01 |
| CLIC2 | 0.001 | 0.01 |
| PLEKHB1 | 0.001 | 0.01 |
| SPAG7 | 0.001 | 0.01 |
| ZNF236 | 0.001 | 0.01 |
| SAMHD1 | 0.001 | 0.01 |
| LAMA2 | 0.001 | 0.01 |
| ERH | 0.001 | 0.01 |
| PSMC3 | 0.001 | 0.01 |
| RIDA | 0.001 | 0.01 |
| RPS15 | 0.001 | 0.01 |
| COQ3 | 0.001 | 0.01 |
| SEPTIN10 | 0.001 | 0.01 |
| THOC1 | 0.001 | 0.01 |
| TOB1 | 0.001 | 0.01 |
| ZNF292 | 0.001 | 0.01 |
| RSU1 | 0.001 | 0.01 |
| RPS15A | 0.001 | 0.01 |
| PAPSS2 | 0.001 | 0.01 |
| TAF2 | 0.001 | 0.01 |
| ZNF764 | 0.001 | 0.01 |
| NUDT9 | 0.001 | 0.01 |
| ABHD18 | 0.001 | 0.01 |
| SYF2 | 0.001 | 0.01 |
| DNAJB5 | 0.001 | 0.01 |
| CXCL2 | 0.001 | 0.01 |
| HHEX | 0.001 | 0.01 |
| PDE4B | 0.001 | 0.01 |
| TBCA | 0.001 | 0.01 |
| RASIP1 | 0.001 | 0.01 |
| EIF2S2 | 0.001 | 0.01 |
| LAMC1 | 0.001 | 0.01 |
| NME6 | 0.001 | 0.01 |
| PFKP | 0.001 | 0.01 |
| MOB1A | 0.001 | 0.01 |
| SIDT2 | 0.001 | 0.011 |
| TMEM33 | 0.001 | 0.011 |
| SORT1 | 0.001 | 0.011 |
| DYSF | 0.001 | 0.011 |
| MUL1 | 0.001 | 0.011 |
| MAPKAPK2 | 0.001 | 0.011 |
| PBX3 | 0.001 | 0.011 |
| MAF | 0.001 | 0.011 |
| DMWD | 0.001 | 0.011 |
| PITRM1 | 0.001 | 0.011 |
| SLC29A1 | 0.001 | 0.011 |
| CTDSPL | 0.001 | 0.011 |
| UBE3B | 0.001 | 0.011 |
| RPS6KA4 | 0.001 | 0.011 |
| TUBGCP2 | 0.001 | 0.011 |
| CTDNEP1 | 0.001 | 0.011 |
| CIR1 | 0.001 | 0.011 |
| XRCC5 | 0.001 | 0.011 |
| SART1 | 0.001 | 0.011 |
| NLGN1 | 0.001 | 0.011 |
| AAGAB | 0.001 | 0.011 |
| AP2B1 | 0.001 | 0.011 |
| COG7 | 0.001 | 0.011 |
| MFGE8 | 0.001 | 0.011 |
| COX6A2 | 0.001 | 0.011 |
| WDR61 | 0.001 | 0.011 |
| COX8A | 0.001 | 0.011 |
| ELF1 | 0.001 | 0.011 |
| VIPAS39 | 0.001 | 0.011 |
| XKR8 | 0.001 | 0.011 |
| ANXA2 | 0.001 | 0.011 |
| SCYL3 | 0.001 | 0.011 |
| CDC16 | 0.001 | 0.011 |
| RXRG | 0.001 | 0.011 |
| HMGB2 | 0.001 | 0.011 |
| UBE2J1 | 0.001 | 0.011 |
| GUSB | 0.001 | 0.011 |
| SCAMP5 | 0.001 | 0.011 |
| C1D | 0.001 | 0.011 |
| DNAAF2 | 0.001 | 0.011 |
| DZIP1 | 0.001 | 0.011 |
| DCTD | 0.001 | 0.011 |
| FBXO7 | 0.001 | 0.011 |
| RALY | 0.001 | 0.012 |
| PRCP | 0.001 | 0.012 |
| RNF115 | 0.001 | 0.012 |
| SURF1 | 0.001 | 0.012 |
| ING2 | 0.001 | 0.012 |
| SDAD1 | 0.001 | 0.012 |
| DUSP26 | 0.001 | 0.012 |
| YLPM1 | 0.001 | 0.012 |
| SEMA4C | 0.001 | 0.012 |
| GIGYF2 | 0.001 | 0.012 |
| NUDT6 | 0.001 | 0.012 |
| DIPK1A | 0.001 | 0.012 |
| RTN1 | 0.001 | 0.012 |
| PDAP1 | 0.001 | 0.012 |
| NCL | 0.001 | 0.012 |
| ACADVL | 0.001 | 0.012 |
| PREB | 0.001 | 0.012 |
| TXNL4A | 0.001 | 0.012 |
| SSH1 | 0.001 | 0.012 |
| SMARCC2 | 0.001 | 0.012 |
| WWP1 | 0.001 | 0.012 |
| HTATIP2 | 0.001 | 0.012 |
| CHCHD7 | 0.001 | 0.012 |
| LARP4B | 0.001 | 0.012 |
| ACTR3 | 0.002 | 0.012 |
| CD47 | 0.002 | 0.012 |
| MRPL39 | 0.002 | 0.012 |
| CCNG1 | 0.002 | 0.012 |
| CCL8 | 0.002 | 0.012 |
| NDUFV1 | 0.002 | 0.012 |
| TRIM23 | 0.002 | 0.012 |
| FEZ1 | 0.002 | 0.013 |
| DSE | 0.002 | 0.013 |
| PTGER4 | 0.002 | 0.013 |
| SNX5 | 0.002 | 0.013 |
| MRPL57 | 0.002 | 0.013 |
| POLE3 | 0.002 | 0.013 |
| NCK1 | 0.002 | 0.013 |
| MRPL48 | 0.002 | 0.013 |
| AARS1 | 0.002 | 0.013 |
| PTPRO | 0.002 | 0.013 |
| HOOK2 | 0.002 | 0.013 |
| CSTF1 | 0.002 | 0.013 |
| VAMP1 | 0.002 | 0.013 |
| WIPF2 | 0.002 | 0.013 |
| FXYD3 | 0.002 | 0.013 |
| MAFB | 0.002 | 0.013 |
| PREP | 0.002 | 0.013 |
| WDR7 | 0.002 | 0.013 |
| DNAI4 | 0.002 | 0.013 |
| ACTR6 | 0.002 | 0.013 |
| RSF1 | 0.002 | 0.013 |
| HYOU1 | 0.002 | 0.013 |
| CSRP3 | 0.002 | 0.014 |
| FBXW7 | 0.002 | 0.014 |
| CTBS | 0.002 | 0.014 |
| PRSS22 | 0.002 | 0.014 |
| HAND1 | 0.002 | 0.014 |
| DAZAP1 | 0.002 | 0.014 |
| ZNF140 | 0.002 | 0.014 |
| BCL7A | 0.002 | 0.014 |
| RXYLT1 | 0.002 | 0.014 |
| EEF1AKNMT | 0.002 | 0.014 |
| GGNBP2 | 0.002 | 0.014 |
| CPT1A | 0.002 | 0.014 |
| NOTCH3 | 0.002 | 0.014 |
| PPA2 | 0.002 | 0.014 |
| INTS5 | 0.002 | 0.014 |
| FLI1 | 0.002 | 0.014 |
| DDX5 | 0.002 | 0.014 |
| SS18L2 | 0.002 | 0.014 |
| PSMC6 | 0.002 | 0.014 |
| NRBF2 | 0.002 | 0.014 |
| VWA8 | 0.002 | 0.014 |
| ARHGEF2 | 0.002 | 0.014 |
| EFS | 0.002 | 0.014 |
| LSM5 | 0.002 | 0.014 |
| CNOT2 | 0.002 | 0.014 |
| UBN1 | 0.002 | 0.014 |
| PIH1D1 | 0.002 | 0.014 |
| FKBP1A | 0.002 | 0.014 |
| COPZ1 | 0.002 | 0.014 |
| OLA1 | 0.002 | 0.014 |
| LINC01140 | 0.002 | 0.014 |
| SF3B3 | 0.002 | 0.014 |
| RMDN1 | 0.002 | 0.014 |
| SEC14L1 | 0.002 | 0.014 |
| CTSA | 0.002 | 0.014 |
| NAMPT | 0.002 | 0.015 |
| ACTR1B | 0.002 | 0.015 |
| PSMD8 | 0.002 | 0.015 |
| SORBS2 | 0.002 | 0.015 |
| MRPS31 | 0.002 | 0.015 |
| TRA2B | 0.002 | 0.015 |
| GPATCH4 | 0.002 | 0.015 |
| RPS6KA2 | 0.002 | 0.015 |
| TGIF1 | 0.002 | 0.015 |
| GNAI1 | 0.002 | 0.015 |
| MED28 | 0.002 | 0.015 |
| MAP2K4 | 0.002 | 0.015 |
| CLPTM1 | 0.002 | 0.015 |
| PRKAR1B | 0.002 | 0.015 |
| AK4 | 0.002 | 0.015 |
| CREB3L2 | 0.002 | 0.015 |
| GET3 | 0.002 | 0.015 |
| DNAL4 | 0.002 | 0.015 |
| ACYP1 | 0.002 | 0.015 |
| ABLIM1 | 0.002 | 0.015 |
| IDS | 0.002 | 0.015 |
| C3orf14 | 0.002 | 0.015 |
| TINAGL1 | 0.002 | 0.015 |
| COLGALT1 | 0.002 | 0.015 |
| PRKCSH | 0.002 | 0.015 |
| SLC38A7 | 0.002 | 0.015 |
| PTGES2 | 0.002 | 0.016 |
| AMZ2 | 0.002 | 0.016 |
| SERPINB6 | 0.002 | 0.016 |
| OR10H3 | 0.002 | 0.016 |
| ITM2A | 0.002 | 0.016 |
| GIPC2 | 0.002 | 0.016 |
| USF2 | 0.002 | 0.016 |
| PRMT2 | 0.002 | 0.016 |
| CYB561D2 | 0.002 | 0.016 |
| DERL2 | 0.002 | 0.016 |
| ETNPPL | 0.002 | 0.016 |
| HMGN3 | 0.002 | 0.016 |
| NUP88 | 0.002 | 0.016 |
| TRMT2B | 0.002 | 0.016 |
| RSRP1 | 0.002 | 0.016 |
| CIB1 | 0.002 | 0.016 |
| PPP2R5D | 0.002 | 0.016 |
| LXN | 0.002 | 0.017 |
| EML2 | 0.002 | 0.017 |
| NARS2 | 0.002 | 0.017 |
| PTGS1 | 0.002 | 0.017 |
| SIRT3 | 0.002 | 0.017 |
| TMEM97 | 0.002 | 0.017 |
| RETREG3 | 0.002 | 0.017 |
| B4GALT3 | 0.002 | 0.017 |
| ATP6V1A | 0.002 | 0.017 |
| NUP107 | 0.002 | 0.017 |
| RALBP1 | 0.002 | 0.017 |
| SCAND1 | 0.002 | 0.017 |
| AGRN | 0.002 | 0.017 |
| FBXO40 | 0.002 | 0.017 |
| ORC6 | 0.002 | 0.017 |
| SPTB | 0.002 | 0.017 |
| ECM2 | 0.002 | 0.017 |
| RPF1 | 0.002 | 0.017 |
| SGCD | 0.002 | 0.017 |
| LGR4 | 0.002 | 0.017 |
| SPP1 | 0.002 | 0.017 |
| VEZF1 | 0.002 | 0.017 |
| TES | 0.002 | 0.017 |
| ENG | 0.002 | 0.017 |
| NNMT | 0.002 | 0.017 |
| SEC23B | 0.002 | 0.017 |
| RUSF1 | 0.002 | 0.017 |
| MICAL2 | 0.003 | 0.018 |
| MYLK | 0.003 | 0.018 |
| WAPL | 0.003 | 0.018 |
| ACKR3 | 0.003 | 0.018 |
| SDHA | 0.003 | 0.018 |
| AKR1A1 | 0.003 | 0.018 |
| DUS4L | 0.003 | 0.018 |
| ZMIZ1 | 0.003 | 0.018 |
| WTAP | 0.003 | 0.018 |
| PRPF8 | 0.003 | 0.018 |
| RAF1 | 0.003 | 0.018 |
| USP1 | 0.003 | 0.018 |
| RASGRP2 | 0.003 | 0.018 |
| ASB9 | 0.003 | 0.018 |
| NR2F2 | 0.003 | 0.018 |
| BUD31 | 0.003 | 0.018 |
| KDM2A | 0.003 | 0.018 |
| NDUFA8 | 0.003 | 0.018 |
| NDUFS3 | 0.003 | 0.018 |
| GRB10 | 0.003 | 0.018 |
| HPR | 0.003 | 0.018 |
| GLT8D1 | 0.003 | 0.018 |
| MOCOS | 0.003 | 0.018 |
| ZC3H7A | 0.003 | 0.018 |
| SLBP | 0.003 | 0.018 |
| EDNRB | 0.003 | 0.018 |
| BCKDK | 0.003 | 0.018 |
| CTDSP2 | 0.003 | 0.018 |
| SPIN1 | 0.003 | 0.018 |
| FANCL | 0.003 | 0.019 |
| TRAPPC9 | 0.003 | 0.019 |
| MBNL1 | 0.003 | 0.019 |
| PAIP1 | 0.003 | 0.019 |
| TXNDC9 | 0.003 | 0.019 |
| PTPN3 | 0.003 | 0.019 |
| CCDC71 | 0.003 | 0.019 |
| ULK1 | 0.003 | 0.019 |
| PAIP2B | 0.003 | 0.019 |
| EIF2AK2 | 0.003 | 0.019 |
| CTPS2 | 0.003 | 0.019 |
| IL1R1 | 0.003 | 0.019 |
| PFN1 | 0.003 | 0.019 |
| NABP2 | 0.003 | 0.019 |
| KIAA0100 | 0.003 | 0.019 |
| APOD | 0.003 | 0.019 |
| RAD51C | 0.003 | 0.019 |
| ARHGAP6 | 0.003 | 0.019 |
| GMFB | 0.003 | 0.019 |
| WDR44 | 0.003 | 0.019 |
| RARRES1 | 0.003 | 0.019 |
| PHF14 | 0.003 | 0.019 |
| TFCP2 | 0.003 | 0.019 |
| NDUFAF7 | 0.003 | 0.019 |
| MTX2 | 0.003 | 0.019 |
| EML1 | 0.003 | 0.019 |
| MIF | 0.003 | 0.019 |
| SSR2 | 0.003 | 0.019 |
| THBS2 | 0.003 | 0.019 |
| TUBGCP4 | 0.003 | 0.019 |
| UGDH | 0.003 | 0.019 |
| TSPAN32 | 0.003 | 0.019 |
| MARCKS | 0.003 | 0.019 |
| MADD | 0.003 | 0.019 |
| DCTN5 | 0.003 | 0.019 |
| NSA2 | 0.003 | 0.019 |
| TJAP1 | 0.003 | 0.019 |
| THRA | 0.003 | 0.019 |
| GUCY1B1 | 0.003 | 0.019 |
| SSR1 | 0.003 | 0.02 |
| ELK3 | 0.003 | 0.02 |
| IQSEC1 | 0.003 | 0.02 |
| MRPS2 | 0.003 | 0.02 |
| IFITM3 | 0.003 | 0.02 |
| ZNF426 | 0.003 | 0.02 |
| EFL1 | 0.003 | 0.02 |
| GIN1 | 0.003 | 0.02 |
| ACAD10 | 0.003 | 0.02 |
| GRM1 | 0.003 | 0.02 |
| SNTA1 | 0.003 | 0.02 |
| METTL7A | 0.003 | 0.02 |
| ABHD5 | 0.003 | 0.02 |
| ZNF329 | 0.003 | 0.02 |
| BIRC3 | 0.003 | 0.02 |
| ARHGDIA | 0.003 | 0.02 |
| PUM2 | 0.003 | 0.02 |
| MTSS1 | 0.003 | 0.02 |
| USP5 | 0.003 | 0.02 |
| GLUL | 0.003 | 0.02 |
| LPL | 0.003 | 0.02 |
| PDGFA | 0.003 | 0.02 |
| C1orf50 | 0.003 | 0.02 |
| PTP4A1 | 0.003 | 0.02 |
| MAGOH | 0.003 | 0.02 |
| AQP3 | 0.003 | 0.02 |
| ISG20L2 | 0.003 | 0.02 |
| CDK17 | 0.003 | 0.02 |
| STX7 | 0.003 | 0.02 |
| SYNGR2 | 0.003 | 0.021 |
| SNX7 | 0.003 | 0.021 |
| MED15 | 0.003 | 0.021 |
| MYO1C | 0.003 | 0.021 |
| FOXK2 | 0.003 | 0.021 |
| AGK | 0.003 | 0.021 |
| ABCE1 | 0.003 | 0.021 |
| VPS35L | 0.003 | 0.021 |
| MT1HL1 | 0.003 | 0.021 |
| SSPN | 0.003 | 0.021 |
| DR1 | 0.003 | 0.021 |
| DCXR | 0.003 | 0.021 |
| AMPH | 0.003 | 0.021 |
| RAD17 | 0.003 | 0.021 |
| MAGI2 | 0.003 | 0.022 |
| WDR11 | 0.003 | 0.022 |
| SPTLC1 | 0.003 | 0.022 |
| RRAGA | 0.003 | 0.022 |
| MECOM | 0.003 | 0.022 |
| CNOT9 | 0.003 | 0.022 |
| GLRX2 | 0.003 | 0.022 |
| TUBA1B | 0.003 | 0.022 |
| FBXL12 | 0.003 | 0.022 |
| FAM171A1 | 0.004 | 0.022 |
| LRIG1 | 0.004 | 0.022 |
| MCRS1 | 0.004 | 0.022 |
| MYNN | 0.004 | 0.022 |
| INHBC | 0.004 | 0.022 |
| COQ2 | 0.004 | 0.022 |
| PSEN2 | 0.004 | 0.022 |
| PPP2R3A | 0.004 | 0.022 |
| VCPKMT | 0.004 | 0.022 |
| MFAP3 | 0.004 | 0.022 |
| MON2 | 0.004 | 0.022 |
| TYMS | 0.004 | 0.022 |
| RSL24D1 | 0.004 | 0.022 |
| CDIPT | 0.004 | 0.022 |
| SYNM | 0.004 | 0.022 |
| RPL8 | 0.004 | 0.023 |
| SFXN3 | 0.004 | 0.023 |
| AMFR | 0.004 | 0.023 |
| ZBTB16 | 0.004 | 0.023 |
| ATP6AP2 | 0.004 | 0.023 |
| HSD17B11 | 0.004 | 0.023 |
| SUMO3 | 0.004 | 0.023 |
| ZNF83 | 0.004 | 0.023 |
| PIK3IP1 | 0.004 | 0.023 |
| ALDH18A1 | 0.004 | 0.023 |
| KMT2A | 0.004 | 0.023 |
| PRUNE1 | 0.004 | 0.023 |
| USP12 | 0.004 | 0.023 |
| PSMA7 | 0.004 | 0.023 |
| NPTX2 | 0.004 | 0.023 |
| RO60 | 0.004 | 0.023 |
| MFNG | 0.004 | 0.023 |
| RAB6B | 0.004 | 0.023 |
| GPR22 | 0.004 | 0.023 |
| DESI2 | 0.004 | 0.023 |
| MOSPD3 | 0.004 | 0.023 |
| AP3S1 | 0.004 | 0.024 |
| ADGRL2 | 0.004 | 0.024 |
| CDKN1C | 0.004 | 0.024 |
| MRPL2 | 0.004 | 0.024 |
| YWHAE | 0.004 | 0.024 |
| PLP2 | 0.004 | 0.024 |
| NDUFS2 | 0.004 | 0.024 |
| KIFAP3 | 0.004 | 0.024 |
| SIAH2 | 0.004 | 0.024 |
| MEF2A | 0.004 | 0.024 |
| HBEGF | 0.004 | 0.024 |
| FBLN5 | 0.004 | 0.024 |
| TLN2 | 0.004 | 0.024 |
| MARCHF8 | 0.004 | 0.024 |
| PIAS1 | 0.004 | 0.024 |
| PXDC1 | 0.004 | 0.024 |
| MKS1 | 0.004 | 0.024 |
| METTL17 | 0.004 | 0.024 |
| HRH4 | 0.004 | 0.024 |
| SLC2A3 | 0.004 | 0.024 |
| KAT5 | 0.004 | 0.025 |
| RHOT1 | 0.004 | 0.025 |
| CUEDC2 | 0.004 | 0.025 |
| HIBCH | 0.004 | 0.025 |
| SH2B3 | 0.004 | 0.025 |
| NDUFB4 | 0.004 | 0.025 |
| LYZ | 0.004 | 0.025 |
| NOL9 | 0.004 | 0.025 |
| CHUK | 0.004 | 0.025 |
| EIF4E2 | 0.004 | 0.025 |
| MSANTD2 | 0.004 | 0.025 |
| ME3 | 0.004 | 0.025 |
| CADPS2 | 0.004 | 0.025 |
| CDK2AP2 | 0.004 | 0.025 |
| PATZ1 | 0.004 | 0.025 |
| IMPDH1 | 0.004 | 0.025 |
| HLA-DQB1 | 0.004 | 0.025 |
| MSN | 0.004 | 0.025 |
| DPY19L2P2 | 0.004 | 0.025 |
| C1orf216 | 0.004 | 0.025 |
| GABARAP | 0.004 | 0.025 |
| ATF7IP2 | 0.004 | 0.025 |
| MAGED1 | 0.004 | 0.025 |
| TNFRSF10B | 0.004 | 0.026 |
| XRCC6 | 0.004 | 0.026 |
| ILF2 | 0.004 | 0.026 |
| MAPK10 | 0.004 | 0.026 |
| SRP54 | 0.004 | 0.026 |
| DYNC1H1 | 0.004 | 0.026 |
| MGAT5 | 0.004 | 0.026 |
| TGFBR1 | 0.005 | 0.026 |
| UBE2Z | 0.005 | 0.026 |
| RIOX2 | 0.005 | 0.026 |
| SNRK | 0.005 | 0.026 |
| FBXW4 | 0.005 | 0.026 |
| DSPP | 0.005 | 0.026 |
| EOLA1 | 0.005 | 0.026 |
| RAB35 | 0.005 | 0.026 |
| TCEA1 | 0.005 | 0.026 |
| ATP6V0D1 | 0.005 | 0.026 |
| ZFX | 0.005 | 0.026 |
| F13A1 | 0.005 | 0.026 |
| MAPK9 | 0.005 | 0.026 |
| LYST | 0.005 | 0.026 |
| PANK2 | 0.005 | 0.026 |
| PTOV1 | 0.005 | 0.026 |
| SPART | 0.005 | 0.026 |
| TARS1 | 0.005 | 0.027 |
| PLEKHA1 | 0.005 | 0.027 |
| SYN2 | 0.005 | 0.027 |
| SPCS2 | 0.005 | 0.027 |
| SGPP1 | 0.005 | 0.027 |
| GTF3A | 0.005 | 0.027 |
| PCNP | 0.005 | 0.027 |
| KAT2B | 0.005 | 0.028 |
| GLG1 | 0.005 | 0.028 |
| ZCCHC14 | 0.005 | 0.028 |
| TP53BP2 | 0.005 | 0.028 |
| ATMIN | 0.005 | 0.028 |
| ARMT1 | 0.005 | 0.028 |
| SNUPN | 0.005 | 0.028 |
| IST1 | 0.005 | 0.028 |
| MSRB2 | 0.005 | 0.028 |
| TUBG2 | 0.005 | 0.028 |
| PAWR | 0.005 | 0.028 |
| TRIP12 | 0.005 | 0.028 |
| NKTR | 0.005 | 0.028 |
| CFLAR | 0.005 | 0.028 |
| SAE1 | 0.005 | 0.028 |
| HSPA13 | 0.005 | 0.028 |
| ZNF22 | 0.005 | 0.028 |
| GSTK1 | 0.005 | 0.028 |
| TIMP4 | 0.005 | 0.028 |
| RNH1 | 0.005 | 0.029 |
| SLC25A44 | 0.005 | 0.029 |
| HMG20A | 0.005 | 0.029 |
| GALNT1 | 0.005 | 0.029 |
| OPA1 | 0.005 | 0.029 |
| NUP205 | 0.005 | 0.029 |
| FAM214B | 0.005 | 0.029 |
| MMD | 0.005 | 0.029 |
| ABCB7 | 0.005 | 0.029 |
| TMEM140 | 0.005 | 0.029 |
| BCL2L13 | 0.005 | 0.029 |
| ZMIZ2 | 0.005 | 0.029 |
| SIN3B | 0.005 | 0.029 |
| CHST15 | 0.005 | 0.029 |
| ZNF136 | 0.005 | 0.029 |
| HCFC1R1 | 0.005 | 0.029 |
| SLCO3A1 | 0.005 | 0.029 |
| RIPOR1 | 0.005 | 0.029 |
| C20orf27 | 0.005 | 0.029 |
| S100A14 | 0.005 | 0.029 |
| LAPTM4A | 0.005 | 0.029 |
| RING1 | 0.005 | 0.029 |
| RAD1 | 0.005 | 0.029 |
| PDZD2 | 0.005 | 0.029 |
| CHST3 | 0.005 | 0.029 |
| GBF1 | 0.005 | 0.029 |
| ZNF133 | 0.005 | 0.029 |
| GALNT2 | 0.005 | 0.03 |
| SCARF1 | 0.005 | 0.03 |
| CARS1 | 0.005 | 0.03 |
| DEGS1 | 0.005 | 0.03 |
| FOLR1 | 0.006 | 0.03 |
| MANSC1 | 0.006 | 0.03 |
| KIF3A | 0.006 | 0.03 |
| ANP32A | 0.006 | 0.03 |
| POLA1 | 0.006 | 0.03 |
| CLASP1 | 0.006 | 0.03 |
| METTL18 | 0.006 | 0.03 |
| ZNF160 | 0.006 | 0.03 |
| HSPB6 | 0.006 | 0.03 |
| ADAP1 | 0.006 | 0.03 |
| ADCY6 | 0.006 | 0.03 |
| EIF4A3 | 0.006 | 0.03 |
| ZSCAN18 | 0.006 | 0.031 |
| PRR11 | 0.006 | 0.031 |
| ERI3 | 0.006 | 0.031 |
| CKS1B | 0.006 | 0.031 |
| TNRC6B | 0.006 | 0.031 |
| KCNK1 | 0.006 | 0.031 |
| SLC31A1 | 0.006 | 0.031 |
| SLC35A1 | 0.006 | 0.031 |
| PTPA | 0.006 | 0.031 |
| SPAG16 | 0.006 | 0.031 |
| MRPL9 | 0.006 | 0.031 |
| MCFD2 | 0.006 | 0.031 |
| DAP | 0.006 | 0.031 |
| SEC62 | 0.006 | 0.031 |
| CD80 | 0.006 | 0.031 |
| CDK5RAP3 | 0.006 | 0.031 |
| ATP5F1D | 0.006 | 0.031 |
| GAS1 | 0.006 | 0.031 |
| COPG1 | 0.006 | 0.032 |
| DAD1 | 0.006 | 0.032 |
| APPL2 | 0.006 | 0.032 |
| TUBB4B | 0.006 | 0.032 |
| ATP6V0E2 | 0.006 | 0.032 |
| NFATC2IP | 0.006 | 0.032 |
| VTN | 0.006 | 0.032 |
| MRPS30 | 0.006 | 0.032 |
| COL4A6 | 0.006 | 0.032 |
| RNASEH2B | 0.006 | 0.032 |
| NOCT | 0.006 | 0.032 |
| RACGAP1 | 0.006 | 0.032 |
| LPIN1 | 0.006 | 0.032 |
| GTPBP4 | 0.006 | 0.032 |
| CD99 | 0.006 | 0.032 |
| USP4 | 0.006 | 0.032 |
| CLCN4 | 0.006 | 0.032 |
| SSBP1 | 0.006 | 0.033 |
| MICA | 0.006 | 0.033 |
| RAB31 | 0.006 | 0.033 |
| ATP2B1 | 0.006 | 0.033 |
| STK19 | 0.006 | 0.033 |
| TBC1D13 | 0.006 | 0.033 |
| HGS | 0.006 | 0.033 |
| MSL1 | 0.006 | 0.033 |
| TTC19 | 0.006 | 0.033 |
| C11orf58 | 0.006 | 0.034 |
| EIF2B2 | 0.006 | 0.034 |
| SORBS3 | 0.007 | 0.034 |
| TRAPPC2 | 0.007 | 0.034 |
| POLD3 | 0.007 | 0.034 |
| RABEP1 | 0.007 | 0.034 |
| UTP11 | 0.007 | 0.034 |
| NDP | 0.007 | 0.034 |
| SEC13 | 0.007 | 0.034 |
| PRMT3 | 0.007 | 0.034 |
| RAB11FIP2 | 0.007 | 0.034 |
| TBP | 0.007 | 0.034 |
| PXDN | 0.007 | 0.034 |
| EIF3I | 0.007 | 0.034 |
| SYT11 | 0.007 | 0.034 |
| NDUFA10 | 0.007 | 0.034 |
| ZNF232 | 0.007 | 0.034 |
| STK26 | 0.007 | 0.034 |
| ICE2 | 0.007 | 0.034 |
| IQGAP2 | 0.007 | 0.035 |
| ADAMTS5 | 0.007 | 0.035 |
| SP110 | 0.007 | 0.035 |
| MIA3 | 0.007 | 0.035 |
| FDX1 | 0.007 | 0.035 |
| NEDD4 | 0.007 | 0.035 |
| TK2 | 0.007 | 0.035 |
| CYB5A | 0.007 | 0.035 |
| SPRY4 | 0.007 | 0.035 |
| RHOG | 0.007 | 0.035 |
| RAB5B | 0.007 | 0.035 |
| LUZP1 | 0.007 | 0.035 |
| MYO6 | 0.007 | 0.035 |
| ZNF506 | 0.007 | 0.035 |
| OSER1 | 0.007 | 0.035 |
| NAXD | 0.007 | 0.035 |
| NARS1 | 0.007 | 0.035 |
| LPAR6 | 0.007 | 0.035 |
| EIF2AK1 | 0.007 | 0.036 |
| EMP2 | 0.007 | 0.036 |
| ZC3H13 | 0.007 | 0.036 |
| CHTOP | 0.007 | 0.036 |
| GALK2 | 0.007 | 0.036 |
| LGALS3 | 0.007 | 0.036 |
| RPS6KA5 | 0.007 | 0.036 |
| CMTR1 | 0.007 | 0.036 |
| PPIP5K2 | 0.007 | 0.036 |
| TPM4 | 0.007 | 0.036 |
| TRIM21 | 0.007 | 0.036 |
| PER1 | 0.007 | 0.036 |
| MORN3 | 0.007 | 0.036 |
| SLC17A5 | 0.007 | 0.037 |
| DYRK1A | 0.007 | 0.037 |
| MRPL11 | 0.007 | 0.037 |
| LEPROTL1 | 0.008 | 0.037 |
| SFPQ | 0.008 | 0.037 |
| COMT | 0.008 | 0.037 |
| CDYL | 0.008 | 0.037 |
| DAPP1 | 0.008 | 0.037 |
| CACTIN | 0.008 | 0.037 |
| FAHD2A | 0.008 | 0.037 |
| ASCC2 | 0.008 | 0.037 |
| RBM38 | 0.008 | 0.037 |
| FBN1 | 0.008 | 0.037 |
| DPT | 0.008 | 0.037 |
| TSC2 | 0.008 | 0.037 |
| IFRD1 | 0.008 | 0.037 |
| PID1 | 0.008 | 0.037 |
| RNF220 | 0.008 | 0.037 |
| OTUD4 | 0.008 | 0.037 |
| AGPAT2 | 0.008 | 0.037 |
| AFTPH | 0.008 | 0.037 |
| HSD17B4 | 0.008 | 0.037 |
| LRRC20 | 0.008 | 0.037 |
| PEX26 | 0.008 | 0.038 |
| PTPRE | 0.008 | 0.038 |
| PUF60 | 0.008 | 0.038 |
| ADRM1 | 0.008 | 0.038 |
| CEPT1 | 0.008 | 0.038 |
| TMEM243 | 0.008 | 0.038 |
| CIB2 | 0.008 | 0.038 |
| DYNLRB1 | 0.008 | 0.038 |
| RNF167 | 0.008 | 0.038 |
| MRPL44 | 0.008 | 0.038 |
| UQCRC1 | 0.008 | 0.038 |
| PIGB | 0.008 | 0.038 |
| SVIL | 0.008 | 0.038 |
| GNB2 | 0.008 | 0.038 |
| GSK3A | 0.008 | 0.038 |
| NOC3L | 0.008 | 0.039 |
| PCSK5 | 0.008 | 0.039 |
| MTRR | 0.008 | 0.039 |
| CPT2 | 0.008 | 0.039 |
| ARGLU1 | 0.008 | 0.039 |
| C11orf71 | 0.008 | 0.039 |
| NACA | 0.008 | 0.039 |
| TBCE | 0.008 | 0.039 |
| PIK3C2B | 0.008 | 0.039 |
| RBM6 | 0.008 | 0.039 |
| ZFP36L2 | 0.008 | 0.039 |
| SH3BGRL | 0.008 | 0.039 |
| EXD2 | 0.008 | 0.039 |
| TM9SF1 | 0.008 | 0.039 |
| MYO15B | 0.008 | 0.039 |
| TMEM267 | 0.008 | 0.039 |
| CCDC59 | 0.008 | 0.039 |
| MCM2 | 0.008 | 0.039 |
| SUN1 | 0.008 | 0.039 |
| FKBP4 | 0.008 | 0.039 |
| CDH5 | 0.008 | 0.04 |
| ERLIN2 | 0.008 | 0.04 |
| TRAFD1 | 0.008 | 0.04 |
| RABGGTA | 0.008 | 0.04 |
| TMEM184C | 0.008 | 0.04 |
| SLC22A18 | 0.008 | 0.04 |
| SNRPC | 0.008 | 0.04 |
| SPOP | 0.008 | 0.04 |
| RCOR1 | 0.008 | 0.04 |
| DUSP6 | 0.008 | 0.04 |
| BMP5 | 0.009 | 0.04 |
| TPP1 | 0.009 | 0.04 |
| WASF2 | 0.009 | 0.041 |
| LGALS1 | 0.009 | 0.041 |
| KDELR1 | 0.009 | 0.041 |
| IL6R | 0.009 | 0.041 |
| SUZ12 | 0.009 | 0.041 |
| RANBP10 | 0.009 | 0.041 |
| POLD2 | 0.009 | 0.041 |
| ZNF675 | 0.009 | 0.041 |
| SMAD3 | 0.009 | 0.041 |
| ARNT | 0.009 | 0.041 |
| SERPINA3 | 0.009 | 0.041 |
| SQOR | 0.009 | 0.041 |
| GOLGA1 | 0.009 | 0.041 |
| AZI2 | 0.009 | 0.041 |
| ANAPC5 | 0.009 | 0.041 |
| RNF34 | 0.009 | 0.041 |
| CHRDL1 | 0.009 | 0.041 |
| CCND1 | 0.009 | 0.041 |
| NOTCH2 | 0.009 | 0.042 |
| SHANK2 | 0.009 | 0.042 |
| MSMO1 | 0.009 | 0.042 |
| RNF121 | 0.009 | 0.042 |
| POLR2J | 0.009 | 0.042 |
| SETDB1 | 0.009 | 0.042 |
| HMOX2 | 0.009 | 0.042 |
| ZNF268 | 0.009 | 0.042 |
| GGA2 | 0.009 | 0.042 |
| MTMR3 | 0.009 | 0.042 |
| RPLP2 | 0.009 | 0.042 |
| LRPPRC | 0.009 | 0.042 |
| IRF1 | 0.009 | 0.042 |
| SUMO1 | 0.009 | 0.042 |
| MSH3 | 0.009 | 0.042 |
| CTNND2 | 0.009 | 0.042 |
| FLNC | 0.009 | 0.042 |
| POT1 | 0.009 | 0.042 |
| TM9SF2 | 0.009 | 0.042 |
| A2M | 0.009 | 0.042 |
| NPR2 | 0.009 | 0.042 |
| HS1BP3 | 0.009 | 0.042 |
| PIP5K1C | 0.009 | 0.042 |
| RAMP2 | 0.009 | 0.042 |
| ABL1 | 0.009 | 0.042 |
| TNNI3 | 0.009 | 0.042 |
| PIEZO1 | 0.009 | 0.042 |
| CEP162 | 0.009 | 0.043 |
| APOO | 0.009 | 0.043 |
| C1orf54 | 0.009 | 0.043 |
| CPED1 | 0.009 | 0.043 |
| IL33 | 0.01 | 0.043 |
| MED23 | 0.01 | 0.043 |
| LIMD1 | 0.01 | 0.043 |
| CRELD1 | 0.01 | 0.043 |
| RASA4 | 0.01 | 0.043 |
| TMEM185B | 0.01 | 0.043 |
| ACO1 | 0.01 | 0.044 |
| LRRN3 | 0.01 | 0.044 |
| CTSZ | 0.01 | 0.044 |
| NDUFB3 | 0.01 | 0.044 |
| UBE2M | 0.01 | 0.044 |
| CROCCP2 | 0.01 | 0.044 |
| KIZ | 0.01 | 0.044 |
| KCNAB2 | 0.01 | 0.044 |
| CCT3 | 0.01 | 0.044 |
| DCAF7 | 0.01 | 0.044 |
| GABRA4 | 0.01 | 0.044 |
| LACTB2 | 0.01 | 0.044 |
| HYI | 0.01 | 0.045 |
| MAPRE2 | 0.01 | 0.045 |
| RBM22 | 0.01 | 0.045 |
| ZNF410 | 0.01 | 0.045 |
| DDX27 | 0.01 | 0.045 |
| TARS2 | 0.01 | 0.045 |
| TM6SF1 | 0.01 | 0.045 |
| CNNM2 | 0.01 | 0.045 |
| DPY19L1 | 0.01 | 0.045 |
| CCND2 | 0.01 | 0.046 |
| FHIP2B | 0.01 | 0.046 |
| ZNF330 | 0.01 | 0.046 |
| RAP1GAP2 | 0.01 | 0.046 |
| ADI1 | 0.01 | 0.046 |
| AR | 0.01 | 0.046 |
| CS | 0.01 | 0.046 |
| ARL4C | 0.01 | 0.046 |
| IL6 | 0.01 | 0.046 |
| RABAC1 | 0.01 | 0.046 |
| BCHE | 0.01 | 0.046 |
| RPN1 | 0.01 | 0.046 |
| C12orf4 | 0.011 | 0.046 |
| COL4A3 | 0.011 | 0.046 |
| GOT2 | 0.011 | 0.046 |
| TUSC2 | 0.011 | 0.047 |
| RPS11 | 0.011 | 0.047 |
| RIN1 | 0.011 | 0.047 |
| TRIB3 | 0.011 | 0.047 |
| SLC12A6 | 0.011 | 0.047 |
| CPE | 0.011 | 0.047 |
| NDUFB11 | 0.011 | 0.047 |
| FAM131A | 0.011 | 0.047 |
| NKX2-5 | 0.011 | 0.047 |
| PARM1 | 0.011 | 0.047 |
| ISLR | 0.011 | 0.047 |
| TCIM | 0.011 | 0.047 |
| APOL3 | 0.011 | 0.047 |
| CD55 | 0.011 | 0.047 |
| RP2 | 0.011 | 0.048 |
| PHB2 | 0.011 | 0.048 |
| ERGIC2 | 0.011 | 0.048 |
| RESF1 | 0.011 | 0.048 |
| VPS26C | 0.011 | 0.048 |
| CEP112 | 0.011 | 0.048 |
| AVEN | 0.011 | 0.048 |
| SLC25A5 | 0.011 | 0.048 |
| ARHGEF15 | 0.011 | 0.049 |
| TNFAIP8 | 0.011 | 0.049 |
| SREBF2 | 0.011 | 0.049 |
| HBB | 0.011 | 0.049 |
| HIRA | 0.011 | 0.049 |
| CIAO1 | 0.011 | 0.049 |
| KL | 0.011 | 0.049 |
| MED9 | 0.011 | 0.049 |
| P2RX4 | 0.011 | 0.049 |
| EEF1D | 0.011 | 0.049 |
| CLU | 0.011 | 0.049 |
| MAST2 | 0.011 | 0.049 |
| ARHGAP44 | 0.011 | 0.049 |
| HBS1L | 0.011 | 0.049 |
| TMEM176B | 0.012 | 0.049 |
| ZNF302 | 0.012 | 0.049 |
| PHLPP1 | 0.012 | 0.049 |
| ARFGAP3 | 0.012 | 0.049 |
| SLN | 0.012 | 0.049 |

DEGs: differentially expressed genes.
