# Supplementary material for: Integrated genomic analysis defines molecular subgroups in dilated cardiomyopathy and identifies novel biomarkers based on machine learning methods
Source: Front Genet. 2023 Feb 7;14:1050696. doi: 10.3389/fgene.2023.1050696 (PMC9941670; doi:10.3389/fgene.2023.1050696)
Supplement: Supplementary file 6 [file Table3.docx]

**Table 3.** The number of differentially expressed genes by case-control and case-case comparisons and weighted gene co-expression analysis modules in each subgroup.

| Subgroups | The specific genes were compared with the normal group | The specific genes were compared with each subgroup | Modular | |  |
| --- | --- | --- | --- | --- | --- |
| Subgroup 1 | 1236 | 605 | | Red and yellow | |
| Subgroup 2  Subgroup 3 | 1388  2617 | 697  1557 | | Black, blue, green and grey  Pink, turquoise and brown | |
