# Supplementary material for: Integrated genomic analysis defines molecular subgroups in dilated cardiomyopathy and identifies novel biomarkers based on machine learning methods
Source: Front Genet. 2023 Feb 7;14:1050696. doi: 10.3389/fgene.2023.1050696 (PMC9941670; doi:10.3389/fgene.2023.1050696)
Supplement: Supplementary file 7 [file Table4.docx]

**Table S4. Enriched pathways in KEGG analysis for each WGCNA module.**

| **Cluster** | **Description** | **P value** | **Adjust P value** |
| --- | --- | --- | --- |
| brown | Parkinson disease | <0.001 | 0.025 |
| pink | Proteasome | <0.001 | 0.013 |
| pink | Spliceosome | 0.001 | 0.045 |
| pink | Amyotrophic lateral sclerosis | 0.001 | 0.045 |
| red | Ribosome | <0.001 | <0.001 |
| red | Thermogenesis | <0.001 | <0.001 |
| red | Chemical carcinogenesis - reactive oxygen species | <0.001 | <0.001 |
| red | Coronavirus disease - COVID-19 | <0.001 | <0.001 |
| red | Oxidative phosphorylation | <0.001 | <0.001 |
| red | Parkinson disease | <0.001 | <0.001 |
| red | Huntington disease | <0.001 | <0.001 |
| red | Diabetic cardiomyopathy | <0.001 | <0.001 |
| red | Prion disease | <0.001 | <0.001 |
| red | Pathways of neurodegeneration - multiple diseases | <0.001 | <0.001 |
| red | Non-alcoholic fatty liver disease | <0.001 | <0.001 |
| red | Amyotrophic lateral sclerosis | <0.001 | <0.001 |
| red | Alzheimer disease | <0.001 | 0.002 |
| red | Retrograde endocannabinoid signaling | 0.003 | 0.039 |
| turquoise | Huntington disease | <0.001 | <0.001 |
| turquoise | Pathways of neurodegeneration - multiple diseases | <0.001 | 0.001 |
| turquoise | Human papillomavirus infection | <0.001 | 0.002 |
| turquoise | Carbon metabolism | <0.001 | 0.002 |
| turquoise | Amyotrophic lateral sclerosis | <0.001 | 0.003 |
| turquoise | FoxO signaling pathway | <0.001 | 0.003 |
| turquoise | Biosynthesis of amino acids | <0.001 | 0.006 |
| turquoise | AGE-RAGE signaling pathway in diabetic complications | <0.001 | 0.008 |
| turquoise | Oxidative phosphorylation | <0.001 | 0.008 |
| turquoise | Alzheimer disease | <0.001 | 0.008 |
| turquoise | Epithelial cell signaling in Helicobacter pylori infection | <0.001 | 0.008 |
| turquoise | Chemical carcinogenesis - reactive oxygen species | <0.001 | 0.008 |
| turquoise | 2-Oxocarboxylic acid metabolism | <0.001 | 0.008 |
| turquoise | Collecting duct acid secretion | <0.001 | 0.008 |
| turquoise | Phagosome | <0.001 | 0.008 |
| turquoise | Diabetic cardiomyopathy | <0.001 | 0.009 |
| turquoise | Sphingolipid signaling pathway | 0.001 | 0.009 |
| turquoise | Cellular senescence | 0.001 | 0.01 |
| turquoise | Endocrine resistance | 0.001 | 0.01 |
| turquoise | Gap junction | 0.001 | 0.011 |
| turquoise | Apoptosis | 0.001 | 0.012 |
| turquoise | Parkinson disease | 0.001 | 0.012 |
| turquoise | Insulin signaling pathway | 0.001 | 0.012 |
| turquoise | Prolactin signaling pathway | 0.001 | 0.013 |
| turquoise | Central carbon metabolism in cancer | 0.001 | 0.013 |
| turquoise | Autophagy - animal | 0.001 | 0.014 |
| turquoise | Relaxin signaling pathway | 0.001 | 0.014 |
| turquoise | Colorectal cancer | 0.002 | 0.02 |
| turquoise | Spliceosome | 0.002 | 0.021 |
| turquoise | Pancreatic cancer | 0.002 | 0.021 |
| turquoise | Chronic myeloid leukemia | 0.002 | 0.021 |
| turquoise | Prion disease | 0.003 | 0.025 |
| turquoise | Acute myeloid leukemia | 0.003 | 0.025 |
| turquoise | Salmonella infection | 0.003 | 0.028 |
| turquoise | Glutathione metabolism | 0.003 | 0.028 |
| turquoise | Endometrial cancer | 0.003 | 0.028 |
| turquoise | Human T-cell leukemia virus 1 infection | 0.003 | 0.028 |
| turquoise | Spinocerebellar ataxia | 0.004 | 0.03 |
| turquoise | Protein processing in endoplasmic reticulum | 0.004 | 0.032 |
| turquoise | Citrate cycle (TCA cycle) | 0.004 | 0.032 |
| turquoise | Glyoxylate and dicarboxylate metabolism | 0.004 | 0.032 |
| turquoise | Vibrio cholerae infection | 0.004 | 0.032 |
| turquoise | Mitophagy - animal | 0.004 | 0.032 |
| turquoise | Insulin resistance | 0.004 | 0.032 |
| turquoise | Thyroid hormone signaling pathway | 0.005 | 0.033 |
| turquoise | Prostate cancer | 0.005 | 0.034 |
| turquoise | ErbB signaling pathway | 0.005 | 0.034 |
| turquoise | Thiamine metabolism | 0.007 | 0.044 |
| turquoise | Fructose and mannose metabolism | 0.007 | 0.045 |
| yellow | Adherens junction | <0.001 | 0.011 |
| yellow | Cellular senescence | 0.001 | 0.046 |
| yellow | FoxO signaling pathway | 0.001 | 0.046 |
| yellow | Long-term depression | 0.001 | 0.046 |
| yellow | Complement and coagulation cascades | 0.001 | 0.046 |
| yellow | Focal adhesion | 0.001 | 0.048 |
